# Gut Microbiota, Glucose, Lipid, and Water-Electrolyte Metabolism in Children With Nonalcoholic Fatty Liver Disease

**DOI:** 10.3389/fcimb.2021.683743

**Published:** 2021-10-28

**Authors:** Xiongfeng Pan, Atipatsa C. Kaminga, Aizhong Liu, Shi Wu Wen, Miyang Luo, Jiayou Luo

**Affiliations:** ^1^ Xiangya School of Public Health, Central South University, Changsha, China; ^2^ Hunan Provincial Key Laboratory of Clinical Epidemiology, Central South University, Changsha, China; ^3^ Department of Mathematics and Statistics, Mzuzu University, Mzuzu, Malawi; ^4^ OMNI Research Group, Ottawa Hospital Research Institute, Ottawa, ON, Canada; ^5^ Department of Obstetrics and Gynaecology University of Ottawa Faculty of Medicine, Ottawa, ON, Canada; ^6^ School of Epidemiology and Public Health, University of Ottawa Faculty of Medicine, Ottawa, ON, Canada

**Keywords:** gut microbiota, glycolipid metabolism, water-electrolyte metabolism, nonalcoholic fatty liver disease, nonalcoholic steatohepatitis

## Abstract

There is evidence that nonalcoholic fatty liver disease (NAFLD) is affected by gut microbiota, glucose, and lipid. However, the function of water-electrolyte metabolism remains undefined in children with NAFLD. Therefore, the aim of this case-control study was to better understand these interactions. The sample consisted of 75 children, aged between 7 and 16, of whom 25 had nonalcoholic fatty liver (NAFL), 25 had nonalcoholic steatohepatitis (NASH), and 25 were obese and without NAFLD. These groups were matched by age, sex, and body mass index. Data were collected between June, 2019 and December, 2019 at the Hunan Children’s Hospital, in China. Microbiome composition in fecal samples was assessed using 16S ribosomal RNA amplicon sequencing. In the clinical indices, 12 glucose and lipid metabolism indices were included, and six water-electrolyte metabolism indices were included. The results indicated that microbiomes of NAFLD children had lower alpha diversity but higher beta diversity index than the other two groups. Specifically, anti-inflammatory and probiotics abundance (e.g., *Faecalibacterium*, *Akkermansia*, and *Bifidobacterium_adolescentis*) was significantly decreased in NAFLD, whereas the abundance of harmful bacteria (e.g., *Staphylococcaceae*) was increased. Moreover, the abundance of butyrate-producing bacteria (e.g., *Faecalibacterium*, *Roseburia_inulinivorans*, *Roseburia_intestinalis*, and *Coprococcus_comes*) was significantly decreased in NASH. The abundance of these bacteria were associated with glucose, lipid, and water-electrolyte metabolism (e.g., glucose, triglyceride, cholesterol, inorganic salt, total body water, etc.), implying that the NAFLD and its severity were associated with glucose, lipid, and water-electrolyte metabolism dysbiosis. Therefore, these findings suggest that the gut microbiome, especially butyrate-producing bacteria, play an important role in the development of NAFLD in children.

## Introduction

Nonalcoholic fatty liver disease (NAFLD) is one of the most common causes of chronic liver disease in children worldwide (Schwimmer et al., 2019b). NAFLD is a complex progressive clinical–pathologic spectrum disease of the liver that starts as a nonalcoholic fatty liver (NAFL), characterized by simple steatosis, and may progress to nonalcoholic steatohepatitis (NASH), characterized by hepatic injury and inflammation (Pan et al., 2020; Pan et al., 2020b). Moreover, some children with NASH develop liver fibrosis and eventually cirrhosis with its life-threatening complications (Farrell et al., 2018). Although several risk factors, such as genetic polymorphisms, epigenetics, lifestyle, and obesity can explain a small part of the NAFLD pathogenesis, most children with these risk factors do not develop NAFLD (Haukeland et al., 2005; Cabre et al., 2016; Kaur et al., 2016). However, a comprehensive understanding as to why certain children develop NAFLD is lacking, which has spurred multidisciplinary research to better understand the intricate NAFLD pathogenesis.

Recent studies have proposed high blood pressure as a potentially key player in the development of NAFLD (Schwimmer et al., 2014). This is due to the fact that hypertension may lead to altered intrahepatic splanchnic circulation and increased intrahepatic vascular resistance, which could induce NAFL to develop into NASH, cirrhosis and portal hypertension (Aneni et al., 2015). Interestingly, gut-liver axis (GLA) dysfunction (e.g., gut ecosystem dysbiosis, alteration of mucosa permeability, and bacterial overgrowth) may contribute to the rising portal pressure in the earliest stages of NAFL, albeit the mechanism of these changes remains unclear (Baffy, 2019; Schwimmer et al., 2019a).

A recent study suggests that GLA may influence the development of NAFLD and trigger the development of NAFL to NASH (Schwimmer et al., 2019a). Specifically, GLA may affect several putative processes involved in the pathophysiology of NAFLD, including liver and systemic inflammation, choline metabolism, insulin resistance (IR), endotoxemia, host capacity for energy harvest, and water-electrolyte metabolism (Zhu et al., 2013; Del Chierico et al., 2014; Boursier et al., 2016; Pan et al., 2020a; Pan et al., 2021). Also of note in these hypotheses is that gut microbiota-mediated imbalance of water-electrolyte metabolism may play a key role in the progression of NAFL to NASH and early portal hypertension (Baffy, 2019). Thus, understanding the imbalance of water-electrolyte metabolism and GLA and identifying novel microbial molecular targets may yield new strategies for understanding the progression from NAFL to NASH and early portal hypertension.

Data-linking imbalance of water-electrolyte metabolism and GLA dysbiosis to the development and severity of NAFL/NASH in children remain poorly documented. Only a few case series have been reported, generally with small sample sizes (only focus on GLA dysbiosis in NAFLD) and heterogeneous populations [without matching for age-, sex, and body mass index (BMI)] (Zhu et al., 2013; Boursier et al., 2016). Therefore, this study aimed at evaluating GLA dysbiosis in children with NAFLD and in children controls matched by age, sex, and BMI so as to identify bacterial composition and anthropometric and demographic features that discriminate NAFLD children from control children.

## Methods

We conducted this study in accordance with the Declaration of Helsinki and obtained approval from The Hunan Children’s Hospital Ethics Research Committee prior to the implementation of the study. Written informed consent was obtained from parents of the enrolled children. Children with NAFLD, aged between 7 and 16, and obese children without NAFLD (controls) matched by age, sex, and BMI were consecutively enrolled into this study between June and December, 2019 at the Institute of Child Health, Hunan Children’s Hospital (Changsha, China). Ultrasongraphic evaluation, using an ultrasound multifrequency curvilinear 3.5 to 5 MHz probe, was performed on the enrolled children by two expert radiologists to detect the occurrence of fatty liver. In this regard, NAFL was defined as liver steatosis after exclusion of concomitant hepatitis infection, and excessive alcohol consumption (the alcohol consumption >140 g/week for boys and >70 g/week for girls). Furthermore, NASH was diagnosed and subsequently confirmed by pediatric clinicians following differential diagnosis. Patients were excluded if they had a history of chronic inflammatory bowel disease, cirrhosis complications, or they had been treated with steatosis-inducing drugs or antibiotics within the 90 days before inclusion.

### Anthropometric and Demographic Measurements

Anthropometric measurements were performed by trained nurses using standard protocols and calibrated instruments. Weight and height of participants were measured with light clothes and without shoes. Blood pressure was measured using a sphygmomanometer for children. BMI was calculated as weight (in kilograms) divided by square of height (in meters). Waist circumference (WC) was measured by using a nonelastic tape around midway between the lower border of the rib cage and the iliac crest at the end of normal expiration (Pan et al., 2020c). Body composition was assessed using bio-impedance (SHHC Body Composition Analysis, China). In this case, the bio-impedance equipment software used estimates of body components at a molecular level (body water, protein, inorganic salt, basic metabolism, and body fat) and estimates of the other body components at a tissue level on a whole and regional body composition analysis (Pan et al., 2019a). Demographic data were collected using a questionnaire that was designed by the Delphi method.

### Clinical Laboratory Measurements

Fasting venous blood samples were collected from the veins of the subjects using a potassium EDTA tube after 12 h of fasting. In addition, blood collection for measuring biochemical markers was performed. Thus, lipid profile and levels of biochemical markers, such as glucose (Glu), low-density cholesterol (LDL), high-density cholesterol (HDL), triglyceride (TG), cholesterol (Chol), uric acid (UA), insulin, serum C-peptide, hemoglobin (HB), glycosylated hemoglobin (HbA1c), aspartate aminotransferase (AST), and alanine aminotransferase (ALT) were measured using an auto analyzer (Olympus AU5400, Tokyo, Japan). Homeostasis model assessment (HOMA) was calculated by the following formula: HOMA-IR = insulin * Glu/22.5, HOMA-IS = 1/HOMA-IR, HOMA-β = 20 * insulin/(Glu-3.5) (Wallace et al., 2004).

### DNA Extraction, Sequencing, and Quantitative Analysis of the Microbiome

Fecal samples were collected during the time that the demographic measurements were taken on the subjects. Then immediately after collection, fecal samples were frozen at −20°C and transported (packed with dry ice) to the laboratory, where they were kept at a temperature of −80°C. Novogene Bioinformatics Institute (Beijing, China) performed the DNA extraction, sequencing, and analysis of the gut microbiomes. Gut microbiota DNA was isolated from fecal samples using the TIANGEN DNA isolation kit (Novogene Bioinformatics Technology Co., Ltd, Beijing, China), according to the manufacturer’s guidelines. The 16S ribosomal RNA sequencing (16S rRNA) gene, comprising V4 regions, was amplified using the primer pair F515 and R806 (5′-GTGCCAGCMGCCGCGGTAA-3′ and 5′-GGACTACVSGGGTATCTAAT-3′, respectively) (Caporaso et al., 2012). The amplicons from the original DNA fragments were purified, quantified, and pooled at an equimolar ratio based on the method described previously. The sequencing of 16S rRNA was performed on the Illumina MiSeq platform at the Novogene (NovaSeq6000, Beijing, China) of the V4 region (insert size 300 bp, read length 250 bp) (Kozich et al., 2013). For taxonomic assignment, sequence reads were grouped into operational taxonomic units (OTUs) at a sequence similarity level of 97%. The raw sequences were first quality controlled using Qiime (version 1.9) with default parameters (including dereplication, chimera filtering, and read error correction), then grouped into OTUs using Uparse pipeline (v7.0.1001) at a sequence similarity level of 97% (Schloss et al., 2009; Edgar, 2013). For each representative sequence, the Greengenes database (version 13.8) and the reference-based method with SortMeRNA were used to annotate OTUs information (Edgar, 2013). The OTUs information was then normalized by 16S rRNA gene copy number, and strain composition analysis was inferred for each sample (Langille et al., 2013).

### Prediction of Potential Function of Gut Microbiota

To study the potential function of gut microbiota, the Tax4Fun functional prediction analysis was used. Also, the 16S rRNA sequences of prokaryotic whole genome were extracted from the Kyoto Encyclopedia of Genes and Genomes (KEGG) database and SILVA database (high-quality rRNA database), then the similarity of the minimum 16S rRNA sequences of the sequenced samples was analyzed as follows. First, the correction matrix was established using BLASTN algorithm (BLAST Bitscore >1500) based on the KEGG database annotated by UProC and PAUDA. Second, the correction matrix was mapped to the SILVA based on the implementation of the SILVA function annotation. Finally, the function of gut microbiota was predicted using the SILVA sequence as a reference sequence clustered out of the sequenced samples of OTUs to obtain functional annotation information.

### Statistical Analysis

Alpha and beta diversity analyses were performed using Qiime (version 1.9) (Caporaso et al., 2010). A nonparametric Mann-Whitney-Wilcoxon test was used to compare alpha and beta diversity indices between different groups (Pan et al., 2018b). Also, the following analyses were performed: rarefaction curve, rank abundance, species accumulation, correlation heatmap for species abundance, and cluster analysis by unweighted pair-group method with arithmetic mean (UPGMA). On the basis of the microbiome diversity data of different groups, unweighted UniFrac principal coordinates analysis (PCoA) was performed to visualize the relationship between NAFLD and gut microbiota diversity (Li et al., 2015; Khan et al., 2018; Pan et al., 2019b). The between- and within-group difference based on rank of Bray-Curtis distance value were analyzed by ANOSIM analysis using R vegan package. One-way analysis of variance (ANOVA) and *post-hoc* Tukey’s honest significant difference tests for multiple comparisons were performed in SPSS software (version 25.0, IBM Corp, USA) to evaluate differences in anthropometric and demographic data among the three groups (two experimental groups and one control group) (Pan et al., 2018a). Co-occurrence associations among anthropometric data, demographic data, and gut microbiota were explored by computing all pairwise Spearman’s rank coefficients (*r*). Unless otherwise stated, a *p*-value less than 0.05 was considered statistically significant.

## Results

### Patient Characteristics

The sample consisted of 75 children, aged between 7 and 16, of whom 25 had nonalcoholic fatty liver (NAFL), 25 had nonalcoholic steatohepatitis (NASH), and 25 were obese and without NAFLD (the control group). Their anthropometric, demographic, and clinical features are shown in **Table 1** and **Appendix 1**. As regards the clinical indices, there were 12 glucose and lipid metabolism indices (TG, Chol, visceral fat, body fat mass, percent body fat, Glu, HDL-C, LDL-C, HB, HbA1c, insulin in serum, and serum C-peptide), and six water-electrolyte metabolism indices (SBP, DBP, intracellular fluid, extracellular fluid, inorganic salt, and total body water).

**Table 1:** Anthropometric and demographic data in different groups.

	Control	NAFL	NASH
Sex (male/female)	23/2	23/2	23/2
Age (year)	10.08 ± 1.78	10.88 ± 1.88	10.88 ± 2.44
BMI (kg/m^2^)	28.21 ± 1.32	27.46 ± 3.33	28.77 ± 4.50
Systolic blood pressure (mmHg)	120.96 ± 11.06	117.40 ± 11.25	126.68 ± 14.93
Diastolic blood pressure (mmHg)	73.48 ± 11.93	69.20 ± 10.56	72.48 ± 9.93
Intracellular fluid (kg)	16.00 ± 3.04	17.58 ± 4.62	19.3 ± 6.77
Extracellular fluid (kg)	9.99 ± 1.93	10.84 ± 2.76	11.87 ± 4.04
Protein (kg)	6.96 ± 1.30	7.41 ± 1.78	8.05 ± 2.52
Inorganic salt (kg)	2.43 ± 0.51	2.69 ± 0.73	2.85 ± 0.93
Body fa mass (kg)	24.23 ± 3.76	24.30 ± 7.26	26.52 ± 9.94
Basic metabolism (kcal)	1,138.36 ± 145.40	1,203.20 ± 214.57	1,256.72 ± 277.75
Total body water	25.78 ± 5.54	28.31 ± 7.24	30.14 ± 9.39
Muscle mass (kg)	32.93 ± 6.93	35.60 ± 9.85	38.39 ± 12.15
Lean body mass (kg)	35.34 ± 6.82	38.36 ± 9.73	40.76 ± 12.92
Weight (kg)	59.23 ± 9.93	61.91 ± 13.75	61.90 ± 20.52
Visceral fat (cm^2^)	123.32 ± 20.24	118.75 ± 38.14	125.03 ± 42.94
Percent body fat (%)	40.72 ± 3.97	38.88 ± 7.22	38.94 ± 4.59
Waist-hip ratio (%)	0.84 ± 0.05	2.40 ± 7.81	0.86 ± 0.06

BMI, body mass index; NAFL, nonalcoholic fatty liver; NASH, nonalcoholic steatohepatitis.

### Taxonomic Composition of the Gut Microbiota

**Figure 1:**
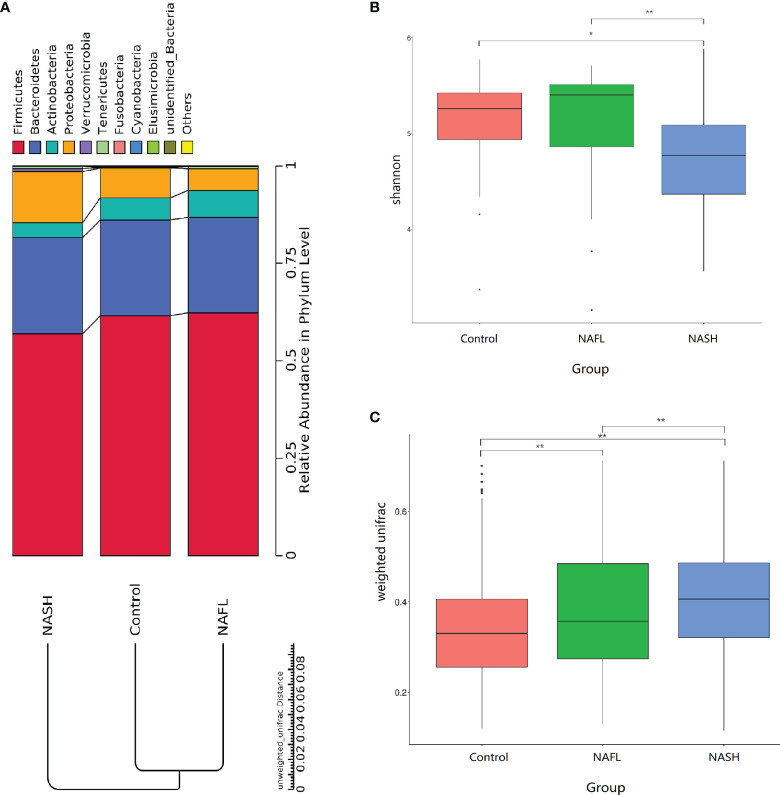
Taxonomic composition of the bacteria. **(A)** The composition and relative abundance of different phyla in different groups (cluster analysis by UPGMA). **(B**, **C)** Alpha and beta diversity indices in different groups. In the analysis of differences between groups in the alpha diversity index (Wilcoxon test based on Shannon Index), the higher the Shannon index, the higher is the species diversity. In the analysis of differences between groups in the beta diversity index (Wilcoxon test based on weighted UniFrac), the higher the sample similarity index within the group, the higher is the level of difference between the different groups. NAFL, nonalcoholic fatty liver; NASH, nonalcoholic steatohepatitis. *p-value < 0.05; **p-value < 0.01.

**Figure 2:**
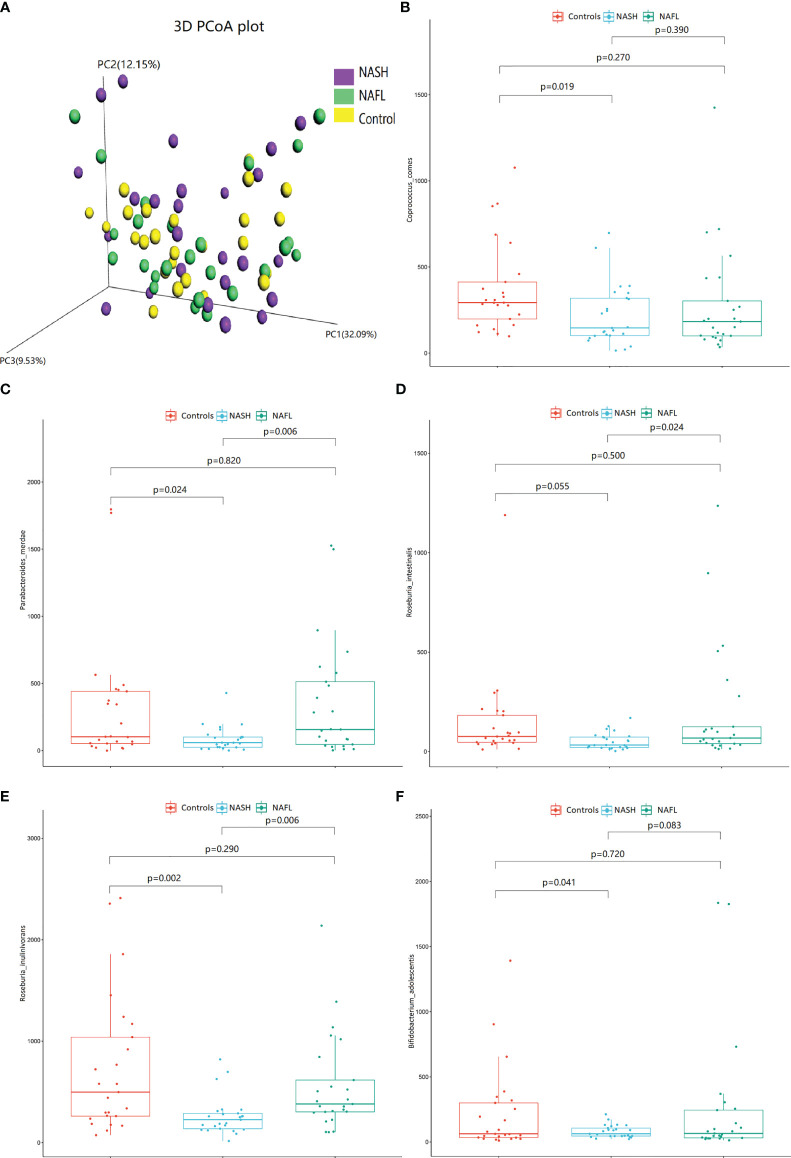
The 3D PCoA plot and scattered dot plot of gut microbiota species in different groups. Unweighted UniFrac 3D PCoA plot **(A)**, PC1 represents one principal component, PC2 and PC3 represent other principal components, and percentage represents the contribution of principal components to sample differences. Each point in the figure represents an individual, and samples in the same group are represented by the same color. Coprococcus_comes **(B)**, Parabacteroides_merdae **(C)**, Roseburia_intestinalis **(D)**, Roseburia_inulinivorans **(E)**, and Bifidobacterium_adolescentis **(F)**. The scattered dot plot of gut microbiota species in different groups are shown. NAFL, nonalcoholic fatty liver; NASH, nonalcoholic steatohepatitis.

With 16S rRNA gene sequencing, 17 phylum, 25 class, 54 order, 109 family, 263 genus, 255 species, and 1333 OTUs were identified. The composition and relative abundance of different phyla in different groups (cluster analysis by UPGMA) are shown in **Figure 1A**. UPGMA cluster analysis showed similar composition of relative abundance between the NAFL and control groups, but this differed from that of the NASH group. The alpha diversity index of the NASH group was significantly lower than that of the NAFL and control groups (**Figure 1B**). However, the beta diversity index of the NASH group was significantly higher than that of the NAFL and control groups, and the NAFL group was significantly higher than control group (**Figure 1C**). Rarefaction curve, rank abundance, and species accumulation box plot, for the three different groups, are shown in **Appendices 2–4**. Unweighted UniFrac PCoA analysis showed that the majority of the samples were clustered by the NAFLD group and control group at the 3D PCoA plot (**Figure 2A**). PC1 represents one principal component, PC2 and PC3 represent other principal components, and percentage represents the contribution of principal components to sample differences. ANOSIM analysis showed the intragroup difference was less than the intergroup difference for the microbiota among NASH-control (*r* = 0.07; *p* = 0.043). However, ANOSIM analysis showed no significant differences among control-NAFL (*r* = −0.01; *p* = 0.595) and NASH-NAFL (*r* = 0.02; *p* = 0.204). In addition, differences in the composition of the bacteria between the cases and controls are shown in **Table 2** and **Appendix 5**. For the NAFL cases and controls, abundance of the Verrucomicrobia at the phylum level; Verrucomicrobiae at the class level; Verrucomicrobiales and Pasteurellales at the order level; three bacteria (e.g., Akkermansiaceae and Pasteurellaceae) at the family level; seven bacteria (e.g., *Akkermansia* and *Haemophilus*) at the genus level; and *Clostridium_ unclassified* at the species level was lower in the cases, whereas abundance of the Enterococcaceae and Staphylococcaceae at the family level and *Novosphingobium* and *Peptoniphilus* at the genus level was lower in the controls. Considering the NASH cases and controls, abundance of the five bacteria (e.g., Rikenellaceae and Barnesiellaceae) at the family level; 11 bacteria (e.g., *Faecalibacterium* and *Agathobacter*) at the genus level; and seven bacteria (e.g., *Coprococcus_comes*, *Roseburia_inulinivorans*, and *Bifidobacterium_adolescentis*) at the species level was lower in the cases, whereas abundance of the Bacillales at the order level and *Acidaminococcus* and *Peptoniphilus* at the genus level was lower in controls. Comparing the NASH and NAFL children, abundance of the three bacteria (e.g., Rikenellaceae and Marinifilaceae) at the family level; 14 bacteria (e.g., *Alistipes and Alloprevotella*) at the genus level; and 7 bacteria (e.g., *Roseburia_inulinivorans* and *Parabacteroides_merdae*) at the species level was lower in the NASH children, whereas abundance of the Bacillales at the family level; six bacteria (e.g., *Gemella* and *Acidaminococcus*) at the genus level; and *Streptococcus_intermedius* at the species level was lower in the NAFL children. Also, many butyrate-producing bacteria (*Faecalibacterium*, *Clostridium_unclassified*, and *Eubacterium_ventriosum* and *Roseburia_intestinalis*) were reduced in the NASH group compared with controls (**Table 2**).

**Table 2:** Gut microbiota in different groups.

Subjects	Phylum	Class	Order	Family	Genus	Species
NAFL versus controls	Verrucomicrobia↓	Verrucomicrobiae↓	Verrucomicrobiale s↓	Akkermansiaceae↓	*Akkermansia*↓	*Clostridium_ unclassified*↓
			Pasteurellales↓	Pasteurellaceae↓	*Haemophilus*↓	
				Enterococcaceae↑	*Cupriavidus*↓	
				Staphylococcaceae↑	*Solobacterium*↓	
				Helicobacteraceae↓	*Helicobacter*↓	
					*Christensenella*↓	
					*Novosphingobium*↑	
					*Peptoniphilus*↑	
					*Muribaculum*↓	
NASH versus controls	NA	NA	Bacillales↑	Rikenellaceae↓Barnesiellaceae↓Peptococcaceae↓Helicobacteraceae↓Nitrosomonadaceae↓	*Faecalibacterium*↓	*Roseburia_inulinivorans* ↓
					*Agathobacter*↓	*Bifidobacterium_adolescentis*↓
					*Roseburia*↓	*Parabacteroides_merdae*↓
					*Lachnospira*↓	*Coprococcus_comes*↓
					*Butyricicoccus*↓	*Eubacterium_ventriosum*↓
					*Barnesiella*↓	*Clostridium_ unclassified*↓
					*Odoribacter*↓	*Odoribacter_splanchnicus*↓
					*Intestinimonas*↓	
					*Eisenbergiella*↓	
					*Acidaminococcus*↑	
					*Helicobacter*↓	
					*Peptoniphilus*↑	
					*Christensenellaceae*↓	
NASH versus NAFL	NA	NA	NA	Rikenellaceae↓	*Citrobacter*↑	*Roseburia_inulinivorans*↓
				Marinifilaceae↓	*Roseburia*↓	*Parabacteroides_merdae*↓
				Peptococcaceae↓	*Alistipes*↓	*Roseburia_intestinalis*↓
				Bacillales↑	*Alloprevotella*↓	*Eubacterium_ventriosum*↓
					*Lachnospira*↓	*Ruminococcus_bicirculans*↓
					*Butyricicoccus*↓	*Ruminococcus_callidus*↓
					*Sellimonas*↓	*Odoribacter_splanchnicus*↓
					*Negativibacillus*↓	*Streptococcus_intermedius*↑
					*Odoribacter*↓	*Bacteroidaceae_bacterium*↓
					*Faecalitalea*↓	
					*Intestinimonas*↓	
					*Eisenbergiella*↓	
					*Morganella*↑	
					*Peptococcus*↓	
					*Marvinbryantia*↓	
					*Gemella*↑	
					*Acidaminococcus*↑	
					*Candidatus_Soleaferrea*↓	
					*Solobacterium*↑	
					*Actinomycetaceae*↑	

NA, not available; NAFL, nonalcoholic fatty liver; NASH, nonalcoholic steatohepatitis.

### Associations Between Glucose, Lipid, and Water-Electrolyte Metabolism and Microbial Community

The scattered dot plot, showing associations of gut microbiota species with glucose, lipid, and water-electrolyte metabolism in different groups, is shown in **Figure 2**. The abundance of *Coprococcus_comes* (**Figure 2B**), *Parabacteroides_merdae* (**Figure 2C**), *Roseburia_intestinalis* (**Figure 2D**), *Roseburia_inulinivorans* (**Figure 2E**), and *Bifidobacterium_adolescentis* (**Figure 2F**) were significantly decreased in NASH patients. The detailed co-occurrence associations between glucose, lipid, and water-electrolyte metabolism (controlled for other anthropometric, demographic, and clinical data) and microbial are listed in **Appendix 6**.

In brief, eight of 12 indices of glucose and lipid (body fat mass, percent body fat, visceral fat, HB, HbA1c, Chol, Glu, and TG) and four of six indices of water-electrolyte (DBP, intracellular fluid, inorganic salt, and total body water) metabolism were significantly associated with microbial community. For water-electrolyte metabolism (**Table 3**), *Haemophilus bacteria* (*Pasteurellales*, *Pasteurellaceae*, and *Haemophilus*) were significantly associated with DBP (*r* = 0.257), body fat mass (*r* = −0.229), and total body water (*r* = −0.237). Furthermore, *Faecalibacterium bacteria* (*Clostridia*, *Eubacteriales*, *Oscillospiraceae*, and *Faecalibacterium*) were significantly associated with intracellular fluid (*r* = −0.242), inorganic salt (*r* = −0.254), and total body water (*r* = −0.229) in the water-electrolyte metabolism. For glucose and lipid metabolism (**Table 3**), *Staphylococcaceae bacteria* (*Bacilli*, *Bacillales*, *Staphylococcaceae*, and *Staphylococcus*) were significantly associated with visceral fat (*r* = 0.239), Chol (*r* = −0.310), and LDL-C (*r* = −0.390). Also, *Akkermansia bacteria* (*Verrucomicrobia*, *Verrucomicrobiae*, *Verrucomicrobiales*, *Akkermansiaceae*, and *Akkermansia*) were significantly associated with TG (*r* = −0.266). Additionally, *Bifidobacterium_adolescentis* bacteria (*Actinomycetia*, *Bifidobacteriales*, *Bifidobacteriaceae*, and *Bifidobacterium*) were significantly associated with TG (*r* = 0.424), Chol (*r* = 0.363), and LDL-C (*r* = 0.372). Moreover, taxonomic composition of the short-chain fatty acid (SCFAs) butyrate-producing bacteria, such as the *Clostridia* and *Bacteroidia*, showed a significant association with glucose and lipid metabolism. Specifically, *Coprococcus comes* bacteria (*Clostridia*, *Eubacteriales*, *Lachnospiraceae*, and *Coprococcus*) were significantly associated with Glu (*r* = −0.472); *Roseburia_inulinivorans* and *Roseburia_intestinalis* bacteria (*Clostridia*, *Eubacteriales*, *Lachnospiraceae*, and *Roseburia*) were significantly associated with body fat percentage (*r* = 0.277), HB (*r* = −0.297), and HbA1c (*r* = −0.302); and *Parabacteroides_merdae* bacteria (*Bacteroidia*, *Bacteroidales*, *Tannerellaceae*, and *Parabacteroides*) were significantly associated with HB (*r* = −0.231), HbA1c (*r* = −0.267). Furthermore, *Alistipes* bacteria (*Bacteroidia*, *Bacteroidales*, *Rikenellaceae*, and *Alistipes*) were significantly associated with visceral fat (*r* = 0.246), body fat percentage (*r* = 0.228), HB (*r* = −0.278), and HbA1c (*r* = −0.239), and *Marinifilaceae* bacteria (*Bacteroidia*, *Marinilabiliales*, *Marinifilaceae*, and *Ancylomarina*) were significantly associated with visceral fat (*r* = 0.251), and body fat percentage (*r* = 0.247).

**Table 3:** Correlation between the glucose, lipid, and water-electrolyte metabolism data and gut microbiota in different groups.

Subjects	Phylum	Class	Order	Family	Genus	Species
NAFL versus controls	Verrucomicrobia↓ (TG *r* = −0.266)	Verrucomicrobiae↓ (TG *r* = −0.266)	Verrucomicrobiales↓	Akkermansiaceae↓	*Akkermansia*↓	NA
			(TG *r* = −0.266)	(TG *r* = −0.266)	(TG *r* = −0.266)	
			Pasteurellales↓ (DBP *r* = 0.257, body fat mass *r* = -0.229, total body water *r* = −0.237)	Pasteurellaceae↓ (DBP *r* = 0.257, body fat mass *r* = −0.229, total body water *r* = −0.237)	*Haemophilus*↓ (DBP *r* = 0.259, body fat mass *r* = −0.230, total body water *r* = −0.239)	
				Staphylococcaceae↑(visceral fat *r* = 0.239, Chol *r* = −0.310, LDL-C *r* =−0.390)		
NASH versus controls	NA	NA	Bacillales↑ (body fat mass *r* = 0.319, visceral fat *r* = 0.325, Chol *r* = −0.271, LDL-C *r* = −0.292)	Rikenellaceae↓ (visceral fat *r* = 0.244, percent body fat *r* = 0.262, HB *r* = −0.268, HbA1c *r* = −0.244)	*Faecalibacterium*↓ (intracellular fluid *r* = −0.242, inorganic salt *r* = −0.254, total body water *r* = −0.229)	*Roseburia_inulinivorans* ↓ (HB *r* = −0.339, HbA1c *r* = −0.237)
					*Roseburia*↓ (HB *r* = −0.359, HbA1c *r* = −0.277)	*Bifidobacterium_adolescentis* ↓ (TG *r* = 0.424, Chol *r* = 0.363, LDL-C *r* = 0.372)
						*Parabacteroides_merdae* ↓ (HB *r* = −0.231, HbA1c *r* = −0.267)
						Coprococcus_comes ↓ (Glu *r* = −0.472)
NASH versus NAFL	NA	NA	NA	Rikenellaceae↓ (visceral fat *r* = 0.244, percent body fat *r* = 0.262, HB *r* = −0.268, HbA1c *r* = −0.244)	*Roseburia*↓ (HB *r* = −0.359, HbA1c *r* = −0.277)	*Roseburia_inulinivorans* ↓ (HB *r* = −0.339, HbA1c *r* = −0.237)
				Marinifilaceae↓ (visceral fat *r* = 0.251, percent body fat *r* = 0.247)	*Alistipes*↓ (visceral fat *r* = 0.246, percent body fat *r* = 0.228, HB *r* = −0.278, HbA1c *r* = −0.239)	*Parabacteroides_merdae* ↓ (HB *r* = -0.231, HbA1c *r* = -0.267)
						*Roseburia_intestinalis* ↓ (percent body fat *r* = 0.277, HB *r* = −0.297, HbA1c *r* = −0.302)

SBP, systolic blood pressure; DBP, diastolic blood pressure; HDL-C, high-density lipoprotein cholesterol; LDL-C, low-density lipoprotein cholesterol; Glu, glucose; TG, triglyceride; Chol, cholesterol; HB, hemoglobin; HbA1c, glycosylated hemoglobin; NA, not available; NAFL, nonalcoholic fatty liver; NASH, nonalcoholic steatohepatitis.

### Potential Functional Prediction of Gut Microbiota

**Figure 3:**
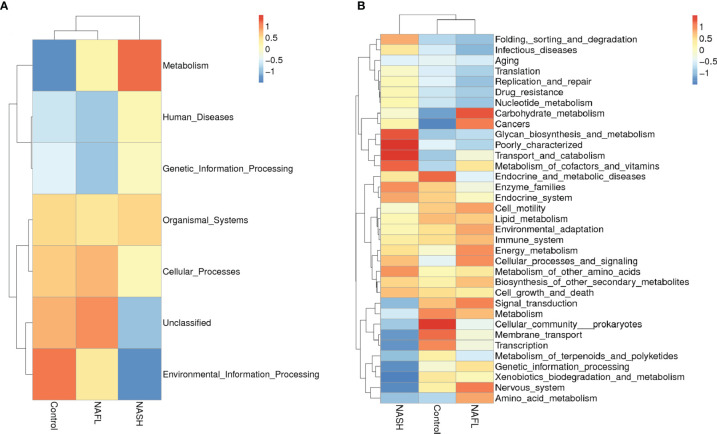
Cluster heatmap of annotated function by Tax4Fun. **(A)** The main functions and **(B)** the metabolism functions. In the figure, the horizontal ordinate represents the sample information and species annotation information; the cluster trees on the left and the top are species clustering and sample clustering, respectively; middle heat map matching is the *Z*-value. *Z*-Value is obtained after standardized treatment of relative abundance of species. It is the difference between the relative abundance of a sample in this classification and the average relative abundance of all samples in this classification divided by the standard deviation of all samples in this classification. NAFL, nonalcoholic fatty liver; NASH, nonalcoholic steatohepatitis.

Tax4Fun functional prediction analysis of the main function of the gut microbiota suggested that the following two predicted functions of the gut microbiota dominated the three subgroups: metabolism and environmental information processing (**Figure 3A**, *p* < 0.05). Tax4Fun functional prediction analysis of the metabolism functions found that most predicted functional categories in KEGG pathways were significantly different among the three subgroups, such as carbohydrate metabolism, glycan biosynthesis and metabolism, transport and catabolism, signal transduction, cellular community prokaryotes, membrane transport, transcription, and amino acid metabolism (**Figure 3B**, *p* < 0.05).

## Discussion

### Gut Dysbiosis and NAFLD

This study examined the association of glucose, lipid, and water-electrolyte metabolism and microbial community with NAFLD in well-characterized obesity in children with and without NAFLD. These results are the same as those of previous studies, indicating that NAFLD children have characteristics of glucose and lipid metabolism disorders, water-electrolyte metabolism disorders, and increased IR unlike their obese counterparts without NAFLD (Aneni et al., 2015; Ji et al., 2019). The contribution of this study is the systematic exploration of the relationship between anthropometric features, demographic characteristics, clinical factors and microbial community, for the first time, in order to reveal the important role that the GLA may play in both the condition of having NAFLD and the severity of NAFLD.

Also, in this study, NAFLD was associated with dysbiosis of the microbiome. Specifically, the NAFLD children were associated with 78 taxonomic composition changes in the bacteria. Thus, compared with previous studies, this study has newly discovered 52 taxonomic composition bacteria with significant differences between the cases and the controls (Pan et al., 2020). Among the studied microflora in different geographic regions, the abundance changes between the cases and the controls were 21 taxonomic composition bacteria, which are the same as in that study, but five taxonomic composition bacteria were the opposite of what that study found. Perhaps these differences may be attributed in part to racial/ethnicity, genetic, diet, and geographical differences (David et al., 2014). For example, high *Prevotella* abundance was linked to fiber-rich diets and high *Bacteroides* abundance was linked to high protein and fat diets (De Filippo et al., 2010; Yatsunenko et al., 2012; David et al., 2014). Interestingly, this study has shown that lower microbial alpha diversity index and higher beta diversity index were significantly associated with NAFLD. Also, in this study, the UPGMA cluster analysis showed similar relative abundance compositions between the NAFL and control groups, but this differed from that in the NASH group. Unweighted UniFrac 3D PCoA plot showed that the majority of the samples were clustered by NASH group and control group, indicating that NASH was a major effect factor for the phylogenetic composition of these samples. Given this observation, we hypothesize that both the condition of having NAFLD and the severity of NAFLD may be explained by a loss of diversity accompanying NAFLD associated microbial abundance dysbiosis.

### Gut Dysbiosis and Glucose, Lipid, and Water-Electrolyte Metabolism

**Figure 4:**
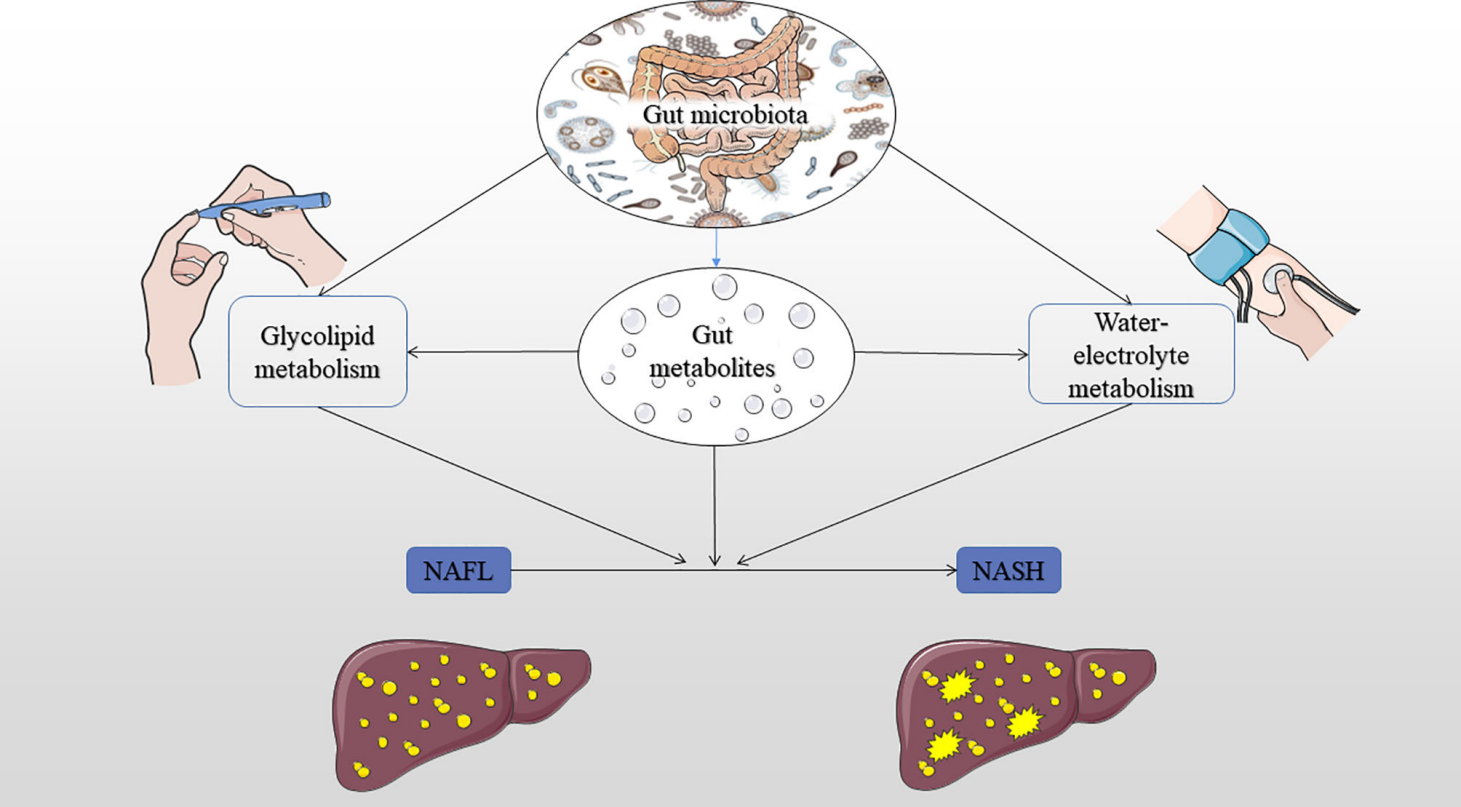
Summary of the hypothesis mechanism process of gut microbiota, glucose, lipid, and water-electrolyte metabolism on NAFLD pathophysiology. IR, insulin resistance; NAFL, nonalcoholic fatty liver; NASH, nonalcoholic steatohepatitis.

This study also showed that the co-occurrence network for the abundance of all microbial taxa correlated with glucose, lipid, and water-electrolyte metabolism. Therefore, we hypothesize that there may be an ecosystem network between the progress of NAFLD and glucose and lipid metabolism or water-electrolyte metabolism and microbial community (**Figure 4**). This hypothesis implies that it may be the ecosystem network dysbiosis, but not the changed relative abundance of a single microbial taxa or indicator, that could be affecting NAFLD. Alternatively, the setting of decreased diversity may affect glucose, lipid, and water-electrolyte metabolism through gut metabolites, then further affect the pathophysiological process of NAFL to NASH development. Another interesting finding of this study was the association between the *Staphylococcaceae*, *Bacillales*, *Rikenellaceae*, *Roseburia*, *Roseburia_inulinivorans*, *Bifidobacterium_adolescentis*, *Parabacteroides_merdae*, *Barnesiellaceae*, *Faecalibacterium* and glucose and lipid metabolism in the occurrence of NAFLD. Also, there was an association between the *Rikenellaceae*, *Roseburia*, *Alistipes*, *Roseburia_inulinivorans*, *Parabacteroides_merdae*, *Roseburia_intestinalis* and glucose and lipid metabolism in the context of the severity of NAFLD. Moreover, there was an association between the *Pasteurellales*, *Pasteurellaceae*, *Haemophilus* and water-electrolyte metabolism in subjects with NAFLD. Worth noting is that these bacteria are all reported for the first time in this study (Baffy, 2019). Therefore, we speculate that these bacteria may play an important role in water-electrolyte metabolism in NAFLD pathogenesis. However, future studies with larger sample sizes may confirm this hypothesis and elucidate the function of these bacteria in NAFLD.

Similarly, these bacteria have been reported in previous studies. For example, some studies found a significantly lower abundance of *Rikenellaceae* and *Parabacteroides* in the NAFLD children than in the control group (Del Chierico et al., 2014; Boursier et al., 2016). Other studies found a significantly lower abundance of *Alistipes*, *Roseburia*, *Bifidobacterium*, *Parabacteroides*, and *Faecalibacterium* in the NASH children (Zhu et al., 2013; Schwimmer et al., 2019a). In addition, several studies have demonstrated that the gut microbiota (e.g., *Clostridium*, *Bifidobacterium*, and *Bacteroides*) affects NAFLD pathogenesis by generating a significant burden for liver-alcohol–scavenging mechanisms (Weimer and Zeikus, 1977; Frantz and McCallum, 1979; Amaretti et al., 2007; Henao-Mejia et al., 2012). In this regard, NAFL may progress to NASH, cirrhosis, and portal hypertension. Furthermore, gut microbiota may contribute to water-electrolyte metabolism disorders and rising portal pressure from the earliest stages of NAFL, although the specific mechanism of these changes remains unclear (Baffy, 2019). A previous study also demonstrated that the abundance of *Bifidobacterium* and *Bacteroides* was greatly reduced in patients with cirrhosis, which contributed to the weakening of the gut barrier due to perhaps the compromised bacterial bile salt hydrolase activity and synthesize deoxycholic acid (Long et al., 2017; Arab et al., 2018). Other cohort study found that the model for endstage liver disease score of patients with cirrhosis had negative correlation with *Ruminococcaceae* and *Rikenellaceae* but had a positive correlation with the abundance of *Staphylococcaceae* (Bajaj et al., 2014). Moreover, GLA metabolic disorders interplay alters bile acid signaling and the release of vasoregulatory gasotransmitters (Chavez-Talavera et al., 2017; Baffy, 2019). These perturbations become prominent in NASH and cirrhosis, increasing the risk of clinically significant water-electrolyte metabolism disorders and portal hypertension, hence, leading to bacterial translocation, sepsis, and liver failure (Lindenmeyer and McCullough, 2018).

### SCFAs-Producing Bacteria Involved in NAFLD

This study also found a lower trend of butyrate-producing bacteria levels in the NASH group compared with controls, suggesting that the SCFAs were potentially involved in the disease development. Anaerobic microorganisms in the colon can ferment soluble dietary fibers to produce volatile fatty acid SCFAs, including acetate, propionate, and butyrate (Li et al., 2021). The role of acetate and propionate in NAFLD is still contradictory. Some previous studies showed that acetate and propionate could maintain low degree of inflammation by inducing proinflammatory T cells through activating GPR41 and GPR43 (e.g., Th1 and Th17) (Kim et al., 2013; Park et al., 2015). Nevertheless, potential roles of acetate and propionate in hepatic lipogenesis, lipid accumulation, and anti-inflammation have been observed in other studies (Nishina and Freedland, 1990; Tedelind et al., 2007). Noteworthy, compelling data from previous experimental models have shown that butyrate can significantly attenuate NASH through thickening of the gut chemical barrier, modulation of gut microbiota, activation of the GLP-1R expression, inhibitory effects of proinflamation, and oxidative damage signaling pathway (Jin et al., 2015; Zhou et al., 2017; Ye et al., 2018; Zhou et al., 2018). Furthermore, taxonomic composition of the butyrate-producing bacteria showed a significant associated with glucose and lipid metabolism, such as the *Clostridia* and *Bacteroidia*. These results may indicate that butyrate-producing bacteria play an important role in the regulation of glucose and lipid metabolism in NAFLD. In a mouse model of NAFLD, induced by high-fat diet, supplementation of sodium butyrate in the diet significantly increased the proportion of *Christensenellaceae*, *Blautia*, and *Lactobacillus* (Zhou et al., 2017; Zhou et al., 2018). Similarly, in a NASH mice model, supplementing sodium butyrate in the diet of mice significantly increased the abundance of *Akkermansia*, *Coprobacillus*, *Coprococcus*, *Delftia*, *Roseburia*, *Sutterella*, and *Coriobacteriaceae*, which reduced the abundance of *Bilophila* and *Rikenellaceae* (Ye et al., 2018). Moreover, *Bilophila* and *Rikenellaceae* have been shown to produce LPS and damage intestinal mucosa, while *Akkermansia*, *Coprobacillus*, *Coprococcus*, and *Roseburia* have the function of preventing immune liver damage related to NASH and improving the protective repair gut barrier (Patterson et al., 2017; Song et al., 2017; Wang et al., 2017).

### Prediction of the Potential Function of the Gut Microbiota

At the function level, functional prediction analysis showed that many bacterial genes involved in carbohydrate metabolism, transport and catabolism, glycan biosynthesis and metabolism, and amino acid metabolism differed significantly between the cases and controls, suggesting that the gut microbiota may affect the absorption of nutrients, which can affect metabolism in the development of NAFLD. By contrast, many normal biological activities of genes of cells linked to signal transduction, cellular community prokaryotes, membrane transport, and transcription were found to be significantly negatively correlated with NASH, suggesting that inflammation produced by gut microbiota may inhibit cell motility.

Specifically, our results have shown that anti-inflammatory and probiotics abundance (e.g., *Faecalibacterium*, *Akkermansia*, and *Bifidobacterium_adolescentis*) were significantly decreased in NAFLD, whereas harmful bacteria abundance (e.g., *Staphylococcaceae*) was increased. Other studies found a significantly lower abundance of *Alistipes*, *Roseburia*, *Bifidobacterium*, *Parabacteroides*, and *Faecalibacterium* in the NASH children (Zhu et al., 2013; Schwimmer et al., 2019a). Furthermore, the abundance of butyrate-producing bacteria Clostridia and Bacteroidia (e.g., *Faecalibacterium*, *Roseburia_inulinivorans*, *Roseburia_intestinalis*, and *Coprococcus_comes*) was significantly decreased in NASH. These bacteria may regulate the metabolism of glucose, lipids, and water electrolytes in NAFLD by producing butyrate (Patterson et al., 2017; Song et al., 2017; Wang et al., 2017). As regards water-electrolyte metabolism, *Haemophilus* bacteria were significantly associated with DBP, body fat mass, and total body water. Additionally, *Faecalibacterium* bacteria were significantly associated with intracellular fluid, inorganic salt, and total body water in the water-electrolyte metabolism. Respecting glucose and lipid metabolism, *Staphylococcaceae* bacteria were significantly associated with visceral fat, Chol, and LDL-C, whereas *Akkermansia* bacteria were significantly associated with TG, and *Bifidobacterium_adolescentis* bacteria were significantly associated with TG, Chol, and LDL-C. Moreover, butyrate-producing bacteria (*Roseburia_inulinivorans* and *Roseburia_intestinalis*) were significantly associated with body fat percentage, HB, and HbA1c. Previous studies found that butyrate can slow down the development of NAFLD. In addition, it can reduce the level of inflammation response, fat metabolism, and glucose metabolism by regulating the gut microbiota (Zhou et al., 2017; Zhou et al., 2018). Specifically, these bacteria may regulate the metabolism of glucose, lipids, and water electrolytes in NAFLD by producing butyrate.

### The Strengths and Limitation of This Study

The discovery of these novel bacteria is very important because it can guide the quest for innovative treatment strategies for NAFLD. For example, in animal studies, reduced abundance of some mucin-degrading bacteria, in obese and diabetic mice, was found to reverse IR, metabolic endotoxemia, and adipose tissue inflammation but controlled inflammation and strengthened the gut barrier (Everard et al., 2013; Zhang et al., 2019). Moreover, previous human studies showed that the probiotic supplementation (e.g., *Lactobacillus acidophilus*, *Bifidobacterium bifidum*, *Lactobacillus rhamnosus*, *Lactobacillus plantarum*, *Lactobacillus salivarius*, *Lactobacillus bulgaricus*, *Lactobacillus casei*, and *Bifidobacterium lactis*) significantly decreased inflammation factors in NAFLD patients (Loguercio et al., 2002; Eslamparast et al., 2014; Pan et al., 2020). Also, GLA was estimated to produce myriads of small molecule metabolites, which may have important physiological and pathological effects on NAFLD, such as leading to disorders of glucose, lipid, and water-electrolyte metabolism. Nevertheless, majority of these metabolic signals in this complex ecosystem need to be further clarified by future research. Furthermore, a recent study has shown that, in addition to probiotic supplements, a new type of synthetic (i.e., smacropore and micropore) activated carbon [Yaq-001 (Yaqrit Ltd.)] can intercept microbial metabolism signals by adsorbing bacterial products, leading to undesirable inflammation effects, which prevent translocation of bacterial products to the GLA (Macnaughtan et al., 2015). However, the preceding novel treatment strategies await additional confirmation before clinical utilization.

There are some limitations related to the findings of this study. First, the study population belonged to only one race, which may limit the generalization of the findings to other races. Therefore, further studies are necessary to elucidate the relationship between race and bacteria in the context of NAFLD. Second, liver biopsy was not performed to classify NAFLD phenotypes because the children included were not eligible for biopsy. Finally, the case-control design does not consider causal inference. Therefore, future studies need to be conducted in larger well-characterized cohorts to validate the hypotheses established in this study and better describe other associations between GLA systematics, glucose and lipid metabolism, water-electrolyte metabolism, and NAFLD phenotypes.

## Conclusions

In summary, the findings of this study suggest an important role of the GLA in NAFLD children. The abundance of anti-inflamatory and probiotics (e.g., *Faecalibacterium*, *Akkermansia*, and *Bifidobacterium_adolescentis*) was significantly decreased in NAFLD, whereas harmful bacteria abundance (e.g., *Staphylococcaceae*) was increased. Moreover, the abundance of butyrate-producing bacteria Clostridia and Bacteroidia (e.g., *Faecalibacterium*, *Roseburia_inulinivorans*, *Roseburia_intestinalis*, and *Coprococcus_comes*) was significantly decreased in NASH. These bacteria were associated with glucose, lipid, and water-electrolyte metabolism (e.g., glucose, triglyceride, cholesterol, inorganic salt, total body water, etc.). Future studies should focus on developing more specific novel therapeutics (e.g., targeted engineered individual microbiota) for NAFLD based on bioengineering technology with high efficacy and specificity.

## Data Availability Statement

The datasets presented in this study can be found in online repositories. The names of the repository/repositories and accession number(s) can be found below: NCBI Sequence Read Archive, PRJNA737039.

## Ethics Statement

The studies involving human participants were reviewed and approved by Central South University. Written informed consent to participate in this study was provided by the participants’ legal guardian/next of kin.

## Author Contributions

XP, AL, and JL contributed to the study design, while AK and JL contributed to the data collection. Statistical analyses and interpretation of results were performed by AK and ML, whereas XP, SW, and JL drafted the manuscript and edited the language. All authors contributed to the article and approved the submitted version.

## Funding

This work was supported by grants from the Chinese Key Project of the National Natural Science Foundation (81872641).

## Conflict of Interest

The authors declare that the research was conducted in the absence of any commercial or financial relationships that could be construed as a potential conflict of interest.

## Publisher’s Note

All claims expressed in this article are solely those of the authors and do not necessarily represent those of their affiliated organizations, or those of the publisher, the editors and the reviewers. Any product that may be evaluated in this article, or claim that may be made by its manufacturer, is not guaranteed or endorsed by the publisher.

## Abbreviations

BMI, body mass index; NAFLD, nonalcoholic fatty liver disease; NAFL, nonalcoholic fatty liver; NASH, nonalcoholic steatohepatitis; BP, blood pressure; LDA, linear discriminant analysis; OTU, operational taxonomic unit; PCR, polymerase chain reaction; rRNA, ribosomal RNA; SCFA, short-chain fatty acid; TG, triglyceride.
