# Supplementary material for: Gut Microbiota, Glucose, Lipid, and Water-Electrolyte Metabolism in Children With Nonalcoholic Fatty Liver Disease
Source: Front Cell Infect Microbiol. 2021 Oct 28;11:683743. doi: 10.3389/fcimb.2021.683743 (PMC8581616; doi:10.3389/fcimb.2021.683743)
Supplement: Supplementary file 1 [file DataSheet_1.doc]

**Supplementary appendix**

**Contents**

**Appendix 1:** Analysis-of-variance (ANOVA) for anthropometric and demographic data in different groups

**Appendix 2:** Rarefaction Curve in different groups

**Appendix 3:** Rank Abundance in different groups

**Appendix 4:** Species accumulation boxplot in different groups

**Appendix 5:** Correlation heatmap for species abundance in different groups. (Phylum (A), Class (B), Order (C), Family (D), and Genus (E))

**Appendix 6:** Correlation between the anthropometric, demographic data and gut microbiota (Phylum (A), Class (B), Order (C), Family (D), and Genus (E) and Species (F))

**Appendix 1: Analysis-of-variance (ANOVA)** for anthropometric and demographic data in different groups

|  | **Control** | **NAFL** | **NASH** | **p** | **p for two groups** | | |
| --- | --- | --- | --- | --- | --- | --- | --- |
|  |  |  |  |  | **Control-NASH** | **NAFL-NASH** | **Control-NAFL** |
| Age, y | 10.08±1.78 | 10.88±1.88 | 10.88±2.44 | 0.288 |  |  |  |
| BMI, kg/m^2^ | 28.21±1.32 | 27.46±3.33 | 28.77±4.50 | 0.995 |  |  |  |
| **Systolic blood pressure, mmHg**↑ | 120.96±11.06 | 117.40±11.25 | 126.68±14.93 | 0.036 |  | 0.011 |  |
| Diastolic blood pressure, mmHg | 73.48±11.93 | 69.20±10.56 | 72.48±9.93 | 0.350 |  |  |  |
| **Intracellular Fluid, kg**↑ | 16.00±3.04 | 17.58±4.62 | 19.3±6.77 | 0.076 | 0.024 |  |  |
| **Extracellular Fluid, kg**↑ | 9.99±1.93 | 10.84±2.76 | 11.87±4.04 | 0.098 | 0.032 |  |  |
| Protein, kg | 6.96±1.30 | 7.41±1.78 | 8.05±2.52 | 0.146 |  |  |  |
| **Inorganic salt, kg**↑ | 2.43±0.51 | 2.69±0.73 | 2.85±0.93 | 0.140 | 0.050 |  |  |
| Body Fat Mass, kg | 24.23±3.76 | 24.30±7.26 | 26.52±9.94 | 0.470 |  |  |  |
| Basic metabolism, kcal | 1138.36±145.40 | 1203.20±214.57 | 1256.72±277.75 | 0.169 |  |  |  |
| **Total body water**↑ | 25.78±5.54 | 28.31±7.24 | 30.14±9.39 | 0.130 | 0.045 |  |  |
| Muscle Mass, kg | 32.93±6.93 | 35.60±9.85 | 38.39±12.15 | 0.156 |  |  |  |
| Lean Body Mass, kg | 35.34±6.82 | 38.36±9.73 | 40.76±12.92 | 0.175 |  |  |  |
| Weight, kg | 59.23±9.93 | 61.91±13.75 | 61.90±20.52 | 0.778 |  |  |  |
| Visceral fat, cm^2^ | 123.32±20.24 | 118.75±38.14 | 125.03±42.94 | 0.808 |  |  |  |
| Percent Body Fat, % | 40.72±3.97 | 38.88±7.22 | 38.94±4.59 | 0.405 |  |  |  |
| Waist-hip ratio, % | 0.84±0.05 | 2.40±7.81 | 0.86±0.06 | 0.377 |  |  |  |
| **ALT, IU/L**↑ | 27.63±13.46 | 35.91±10.97 | 98.43±45.15 | 0.000 | 0.000 | 0.000 |  |
| **AST, IU/L**↑ | 22.61±11.37 | 23.88±8.37 | 47.34±18.50 | 0.000 | 0.000 | 0.000 |  |
| **AST/ALT**↓ | 0.88±0.29 | 0.70±0.13 | 0.49±0.09 | 0.000 | 0.000 | 0.000 | 0.002 |
| TBAC, μmol/L | 3.18±1.46 | 3.82±4.03 | 3.81±2.18 | 0.652 |  |  |  |
| Glucose (Glu), mmol/L | 4.19±0.33 | 4.32±0.37 | 4.77±1.82 | 0.152 |  |  |  |
| **Uric acid (UA), μmol/L**↑ | 340.36±83.35 | 410.28±73.82 | 408.52±67.98 | 0.002 | 0.002 |  | 0.002 |
| Triglyceride (TG), mmol/L | 1.23±0.52 | 1.61±0.95 | 1.55±0.83 | 0.207 |  |  |  |
| Cholesterol (Chol), mmol/L | 4.16±0.60 | 3.85±0.85 | 3.91±0.83 | 0.334 |  |  |  |
| HDL-C, mmol/L | 1.20±0.25 | 1.13±0.21 | 1.11±0.22 | 0.349 |  |  |  |
| LDL-C, mmol/L | 2.39±0.49 | 2.03±0.79 | 2.17±0.65 | 0.162 |  |  |  |
| **HB, g/dl**↑ | 15.40±0.65 | 16.18±1.00 | 16.49±1.13 | 0.000 | 0.000 |  | 0.005 |
| **HbA1c, g/dl**↑ | 0.76±0.06 | 0.85±0.08 | 0.89±0.24 | 0.014 | 0.004 |  |  |
| HbA1c/HB | 4.99±0.34 | 5.25±0.43 | 5.42±1.27 | 0.167 |  |  |  |
| Serum cortisol (CORT), μg/dl | 9.66±5.28 | 8.62±3.15 | 9.58±5.24 | 0.683 |  |  |  |
| **Insulin in serum, μU/mL**↑ | 24.23±11.53 | 28.57±13.61 | 36.87±18.91 | 0.014 | 0.004 |  |  |
| **Serum C-peptide, ng/mL**↑ | 3.13±0.79 | 3.32±1.10 | 4.27±1.39 | 0.001 | 0.001 | 0.004 |  |
| Cu, μg/mL | 0.92±0.12 | 0.96±0.08 | 0.92±0.09 | 0.268 |  |  |  |
| **Zn, μg/mL**↑ | 6.28±0.99 | 6.93±1.14 | 6.51±1.13 | 0.110 |  |  | 0.039 |
| Ca, μg/mL | 61.86±6.76 | 61.36±6.08 | 61.26±6.05 | 0.937 |  |  |  |
| Mg, μg/mL | 37.33±3.70 | 37.39±3.86 | 36.94±2.19 | 0.877 |  |  |  |
| Fe, μg/mL | 385.19±32.77 | 387.45±38.43 | 372.28±52.42 | 0.392 |  |  |  |
| **Pb, μg/L**↓ | 31.84±12.61 | 40.41±19.58 | 27.67±9.28 | 0.009 |  | 0.003 | 0.040 |
| **HOMA-IR**↑ | 4.51±2.17 | 5.47±2.51 | 7.86±4.92 | 0.003 | 0.001 | 0.016 |  |
| HOMA-β | 912.01±605.29 | 880.02±740.64 | 1060.48±986.82 | 0.693 |  |  |  |
| **HOMA-IS**↓ | 0.26±0.11 | 0.23±0.14 | 0.17±0.09 | 0.020 | 0.006 |  |  |

ALT, Alanine aminotransferase; AST, Aspartate aminotransferase; TBAC, Total bile acid; HDL-C, High density lipoprotein cholesterol; LDL-C, Low density lipoprotein cholesterol; HB, Hemoglobin; HbA1c, Glycosylated hemoglobin; HbA1c/HB, Glycosylated hemoglobin ratio; BMI, body mass index; HOMA, Homeostasis model assessment; NAFL, nonalcoholic fatty liver; NASH, non-alcoholic steatohepatitis.

**Appendix 2:** **Rarefaction Curve in different groups**

In the rarefaction curve, the abscissa is the number of sequencing strips randomly selected from a sample, and the ordinate is the number of OTUs that can be constructed based on the number of sequencing strips. It is used to reflect the sequencing depth. Different samples use different color curves Said. When the curve is flat, it means that the amount of sequencing data is gradually reasonable, and more data will only produce a small number of new species (OTUs).


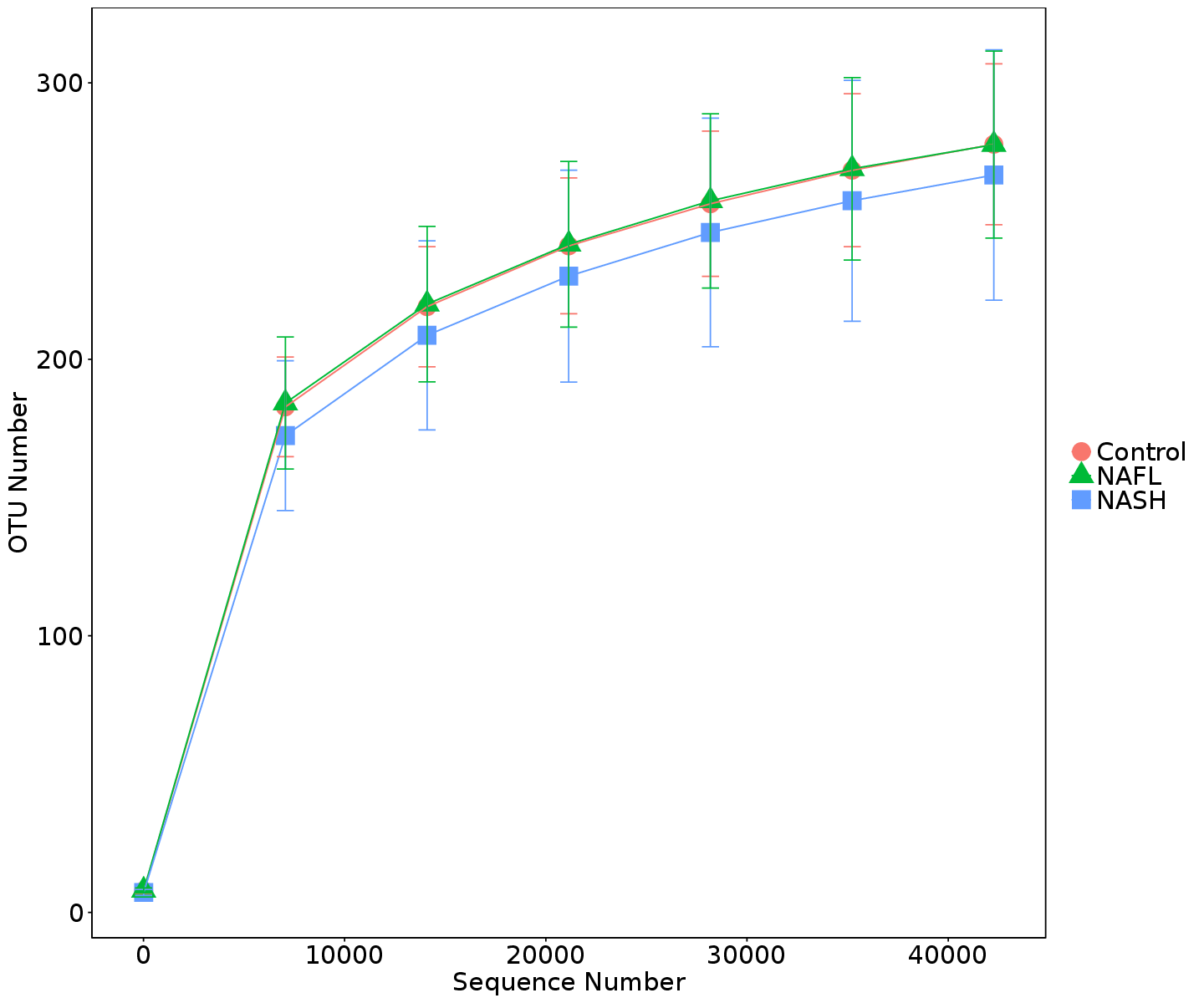


**Appendix 3:** **Rank abundance in different groups**

In the rank abundance curve, the abscissa is the sequence number sorted by the abundance of OTUs, and the ordinate is the relative abundance of the corresponding OTUs. Different samples are represented by polylines of different colors. In the horizontal direction, the richness of the species is reflected by the width of the curve. The higher the richness of the species, the greater the span of the curve on the horizontal axis; in the vertical direction, the smoothness of the curve reflects the species in the sample. The degree of uniformity, the smoother the curve, the more uniform the distribution of species.


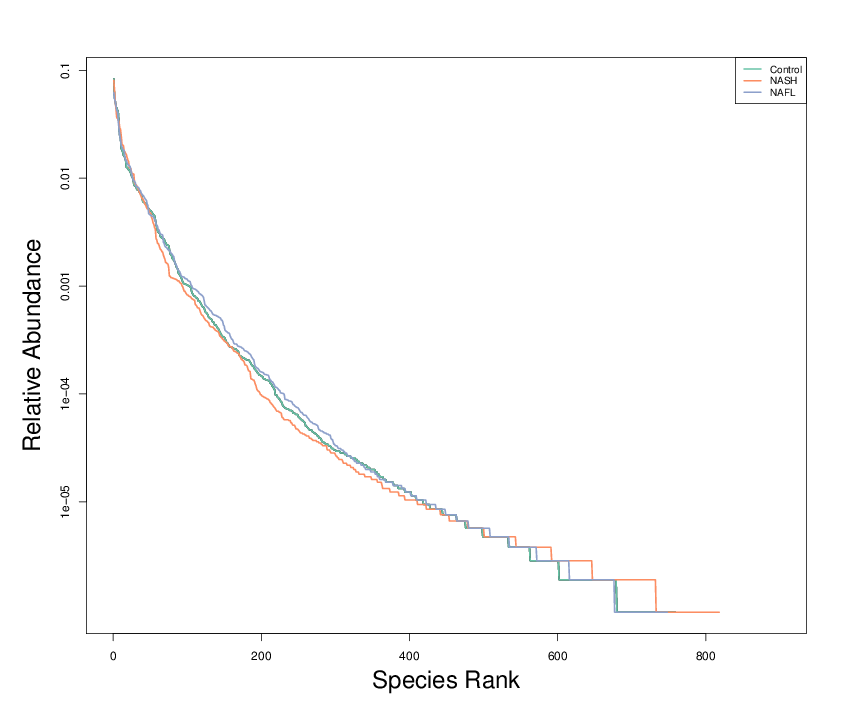


**Appendix 4:** **Species accumulation boxplot in different groups**

The abscissa is the sample size; the ordinate is the OTUs. The results reflect the rate at which new OTUs (new species) appear under continuous sampling. Within a certain range, with the increase of the sample size, if the boxplot shows a sharp rise, it means that a large number of species have been found in the community; when the boxplot tends to be flat, it means the species in this environment It does not increase significantly with the increase in sample size. The boxplot of species accumulation can be used to judge whether the sample size is sufficient. A sharp rise in the position of the boxplot indicates that the sample size is not small, and the sample size needs to be increased; otherwise, it indicates that the sampling is sufficient and data analysis can be performed.


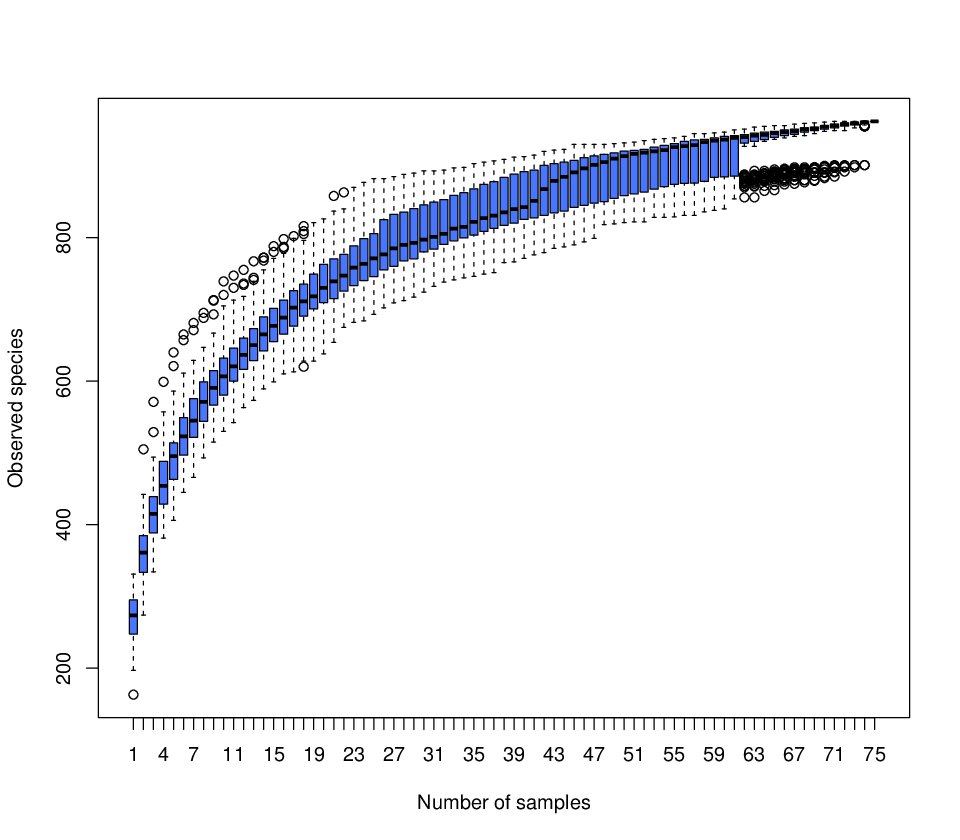


**Appendix 5: Correlation heatmap for species abundance in different groups. (Phylum (A), Class (B), Order (C), Family (D), and Genus (E))**


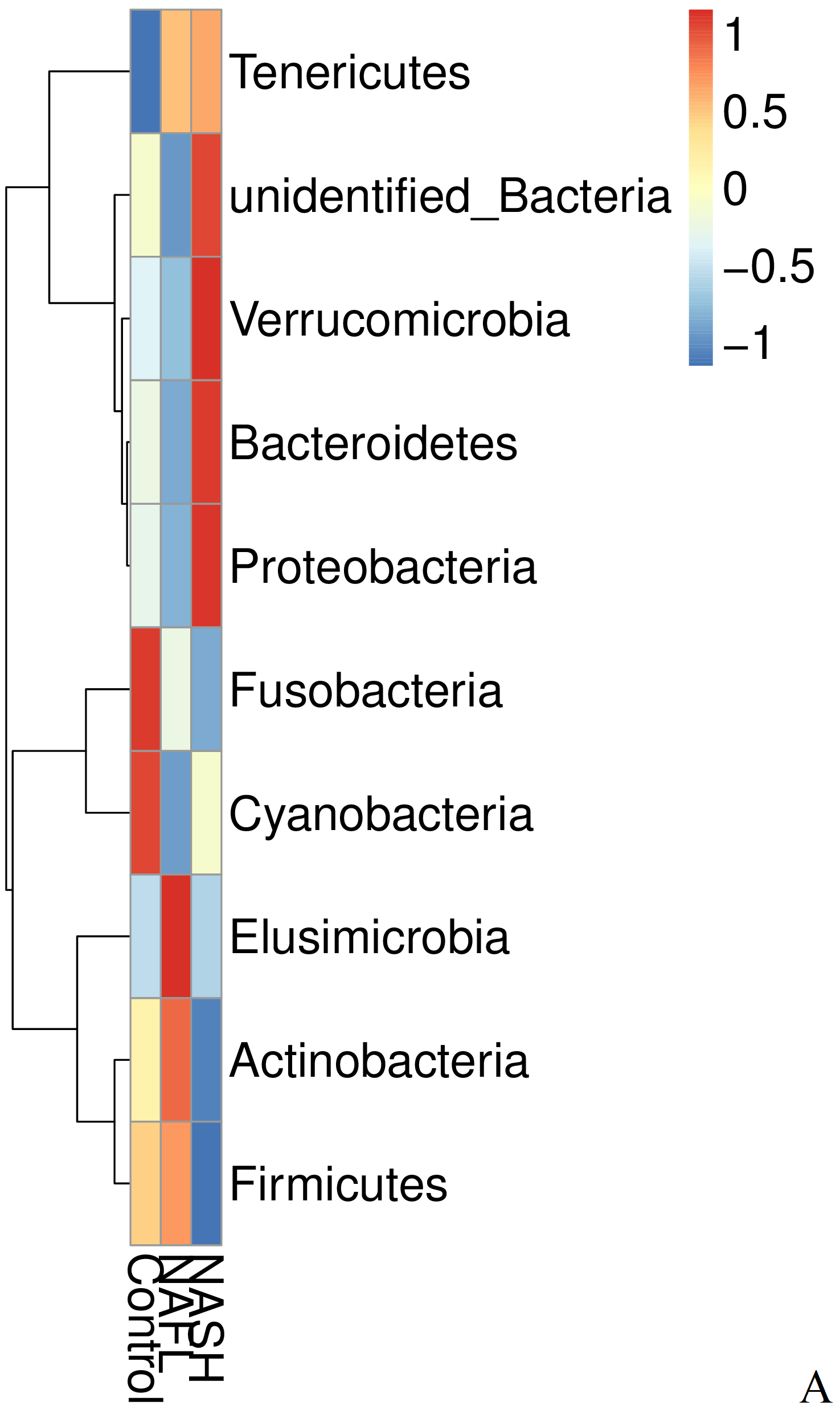


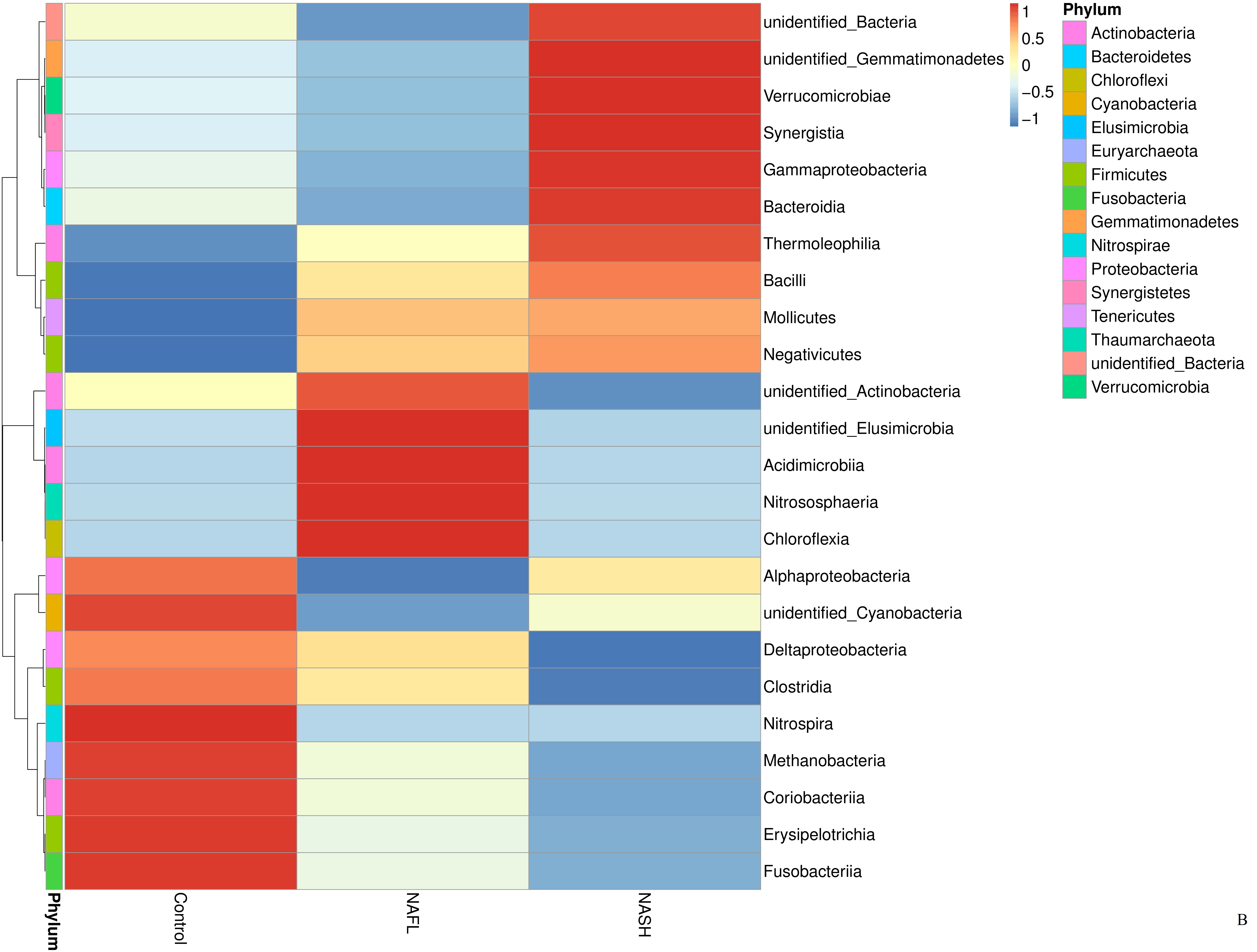


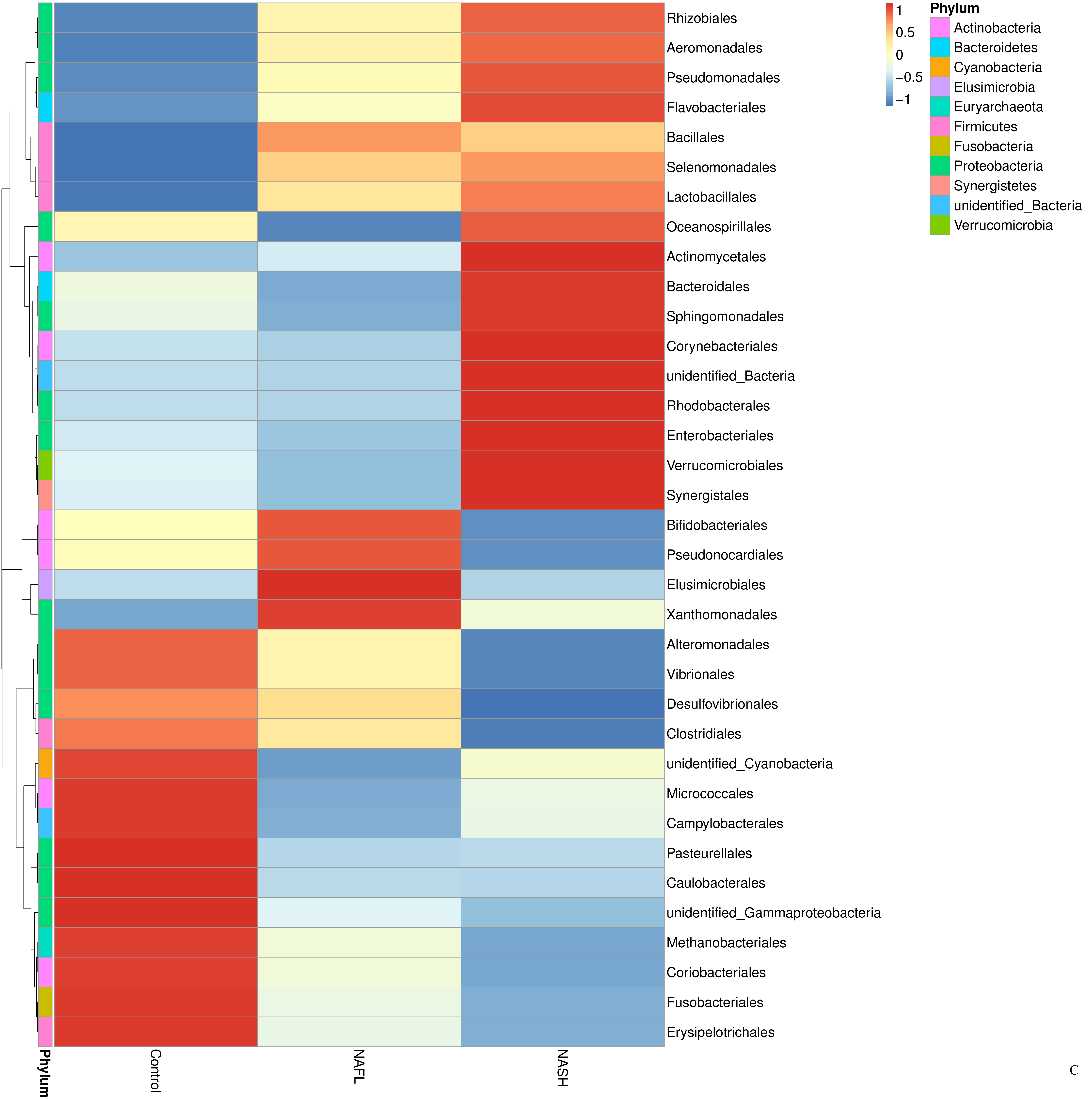


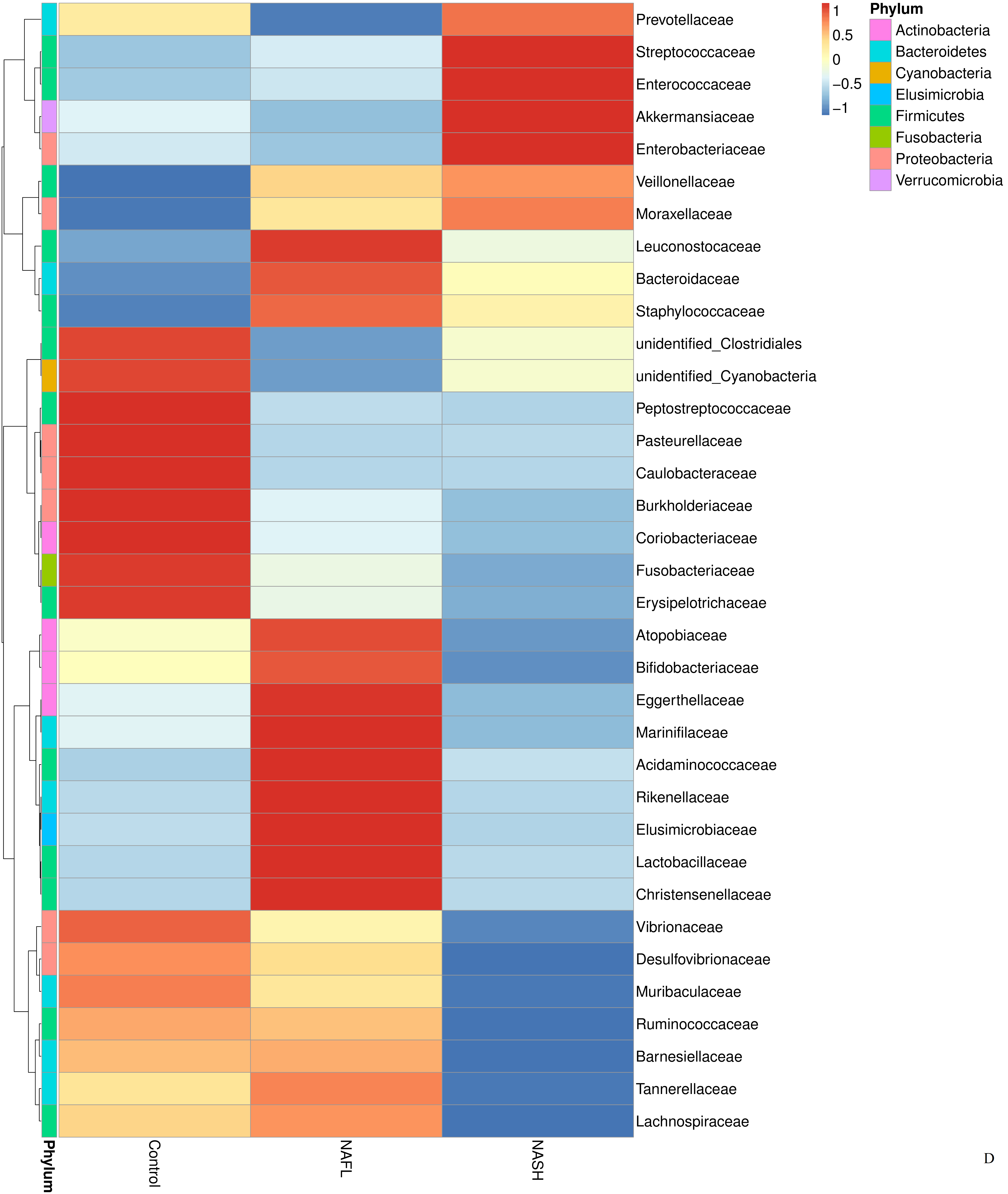


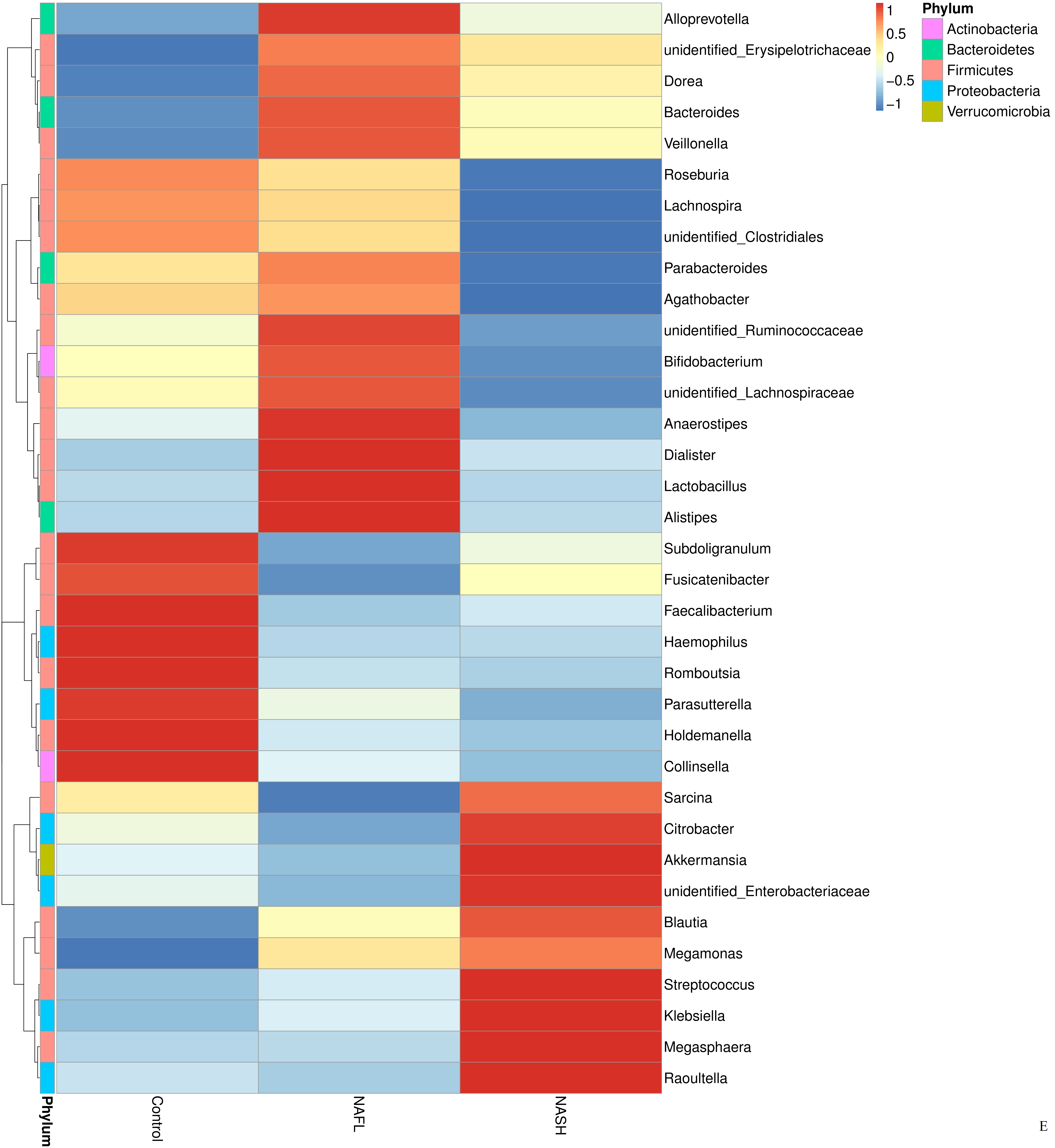


**Appendix 6:** **Correlation between the anthropometric, demographic data and gut microbiota (Phylum (A), Class (B), Order (C), Family (D), and Genus (E) and Species (F))**


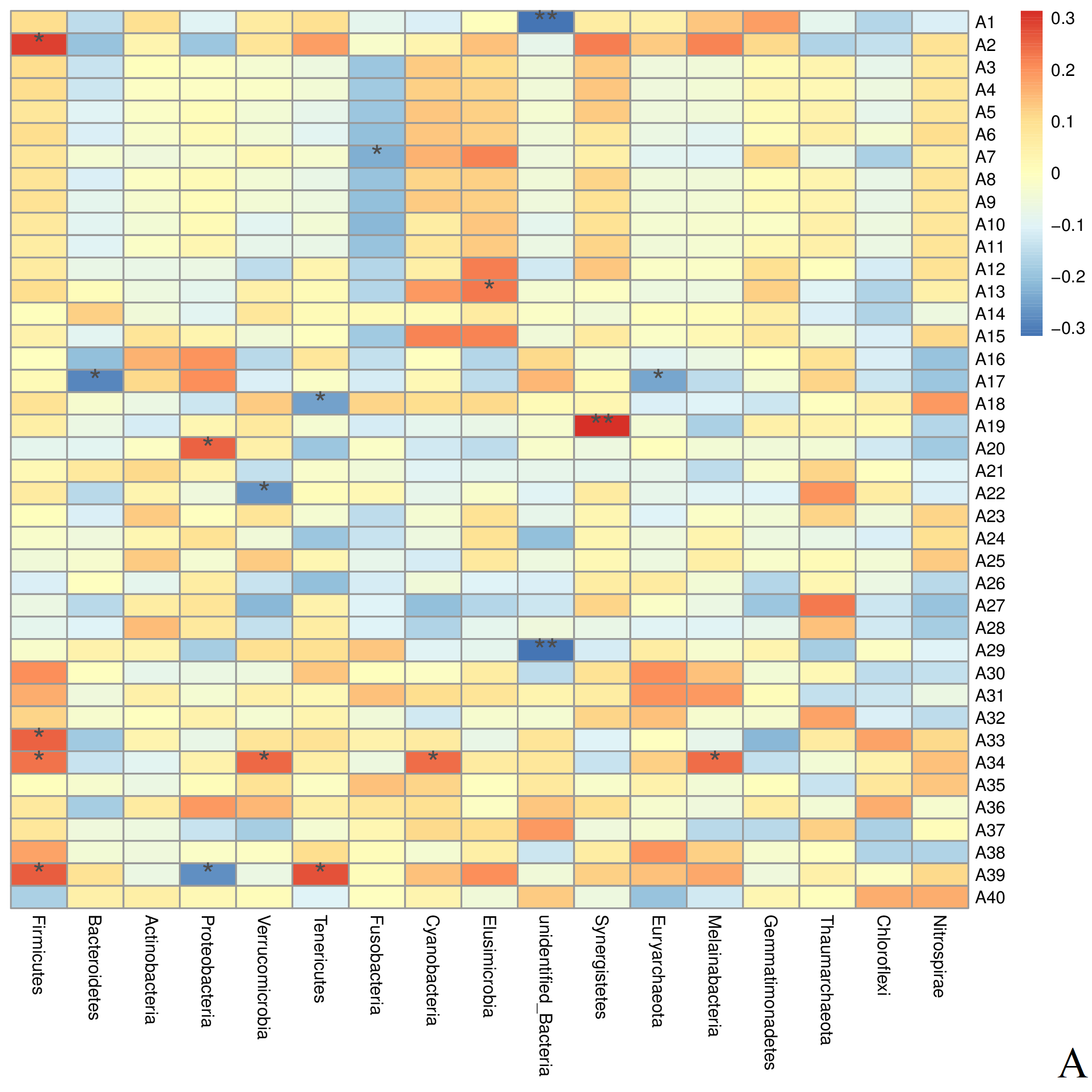


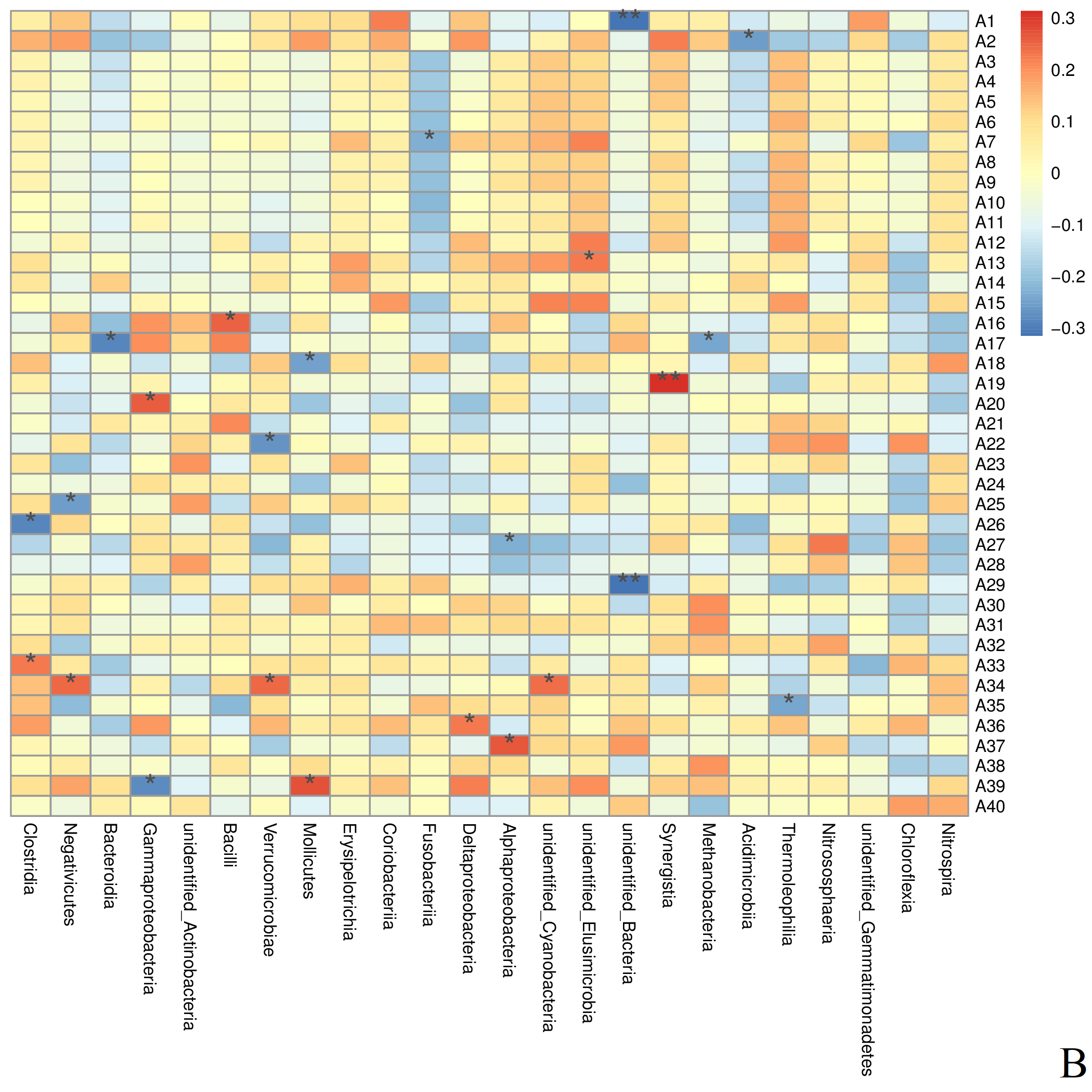

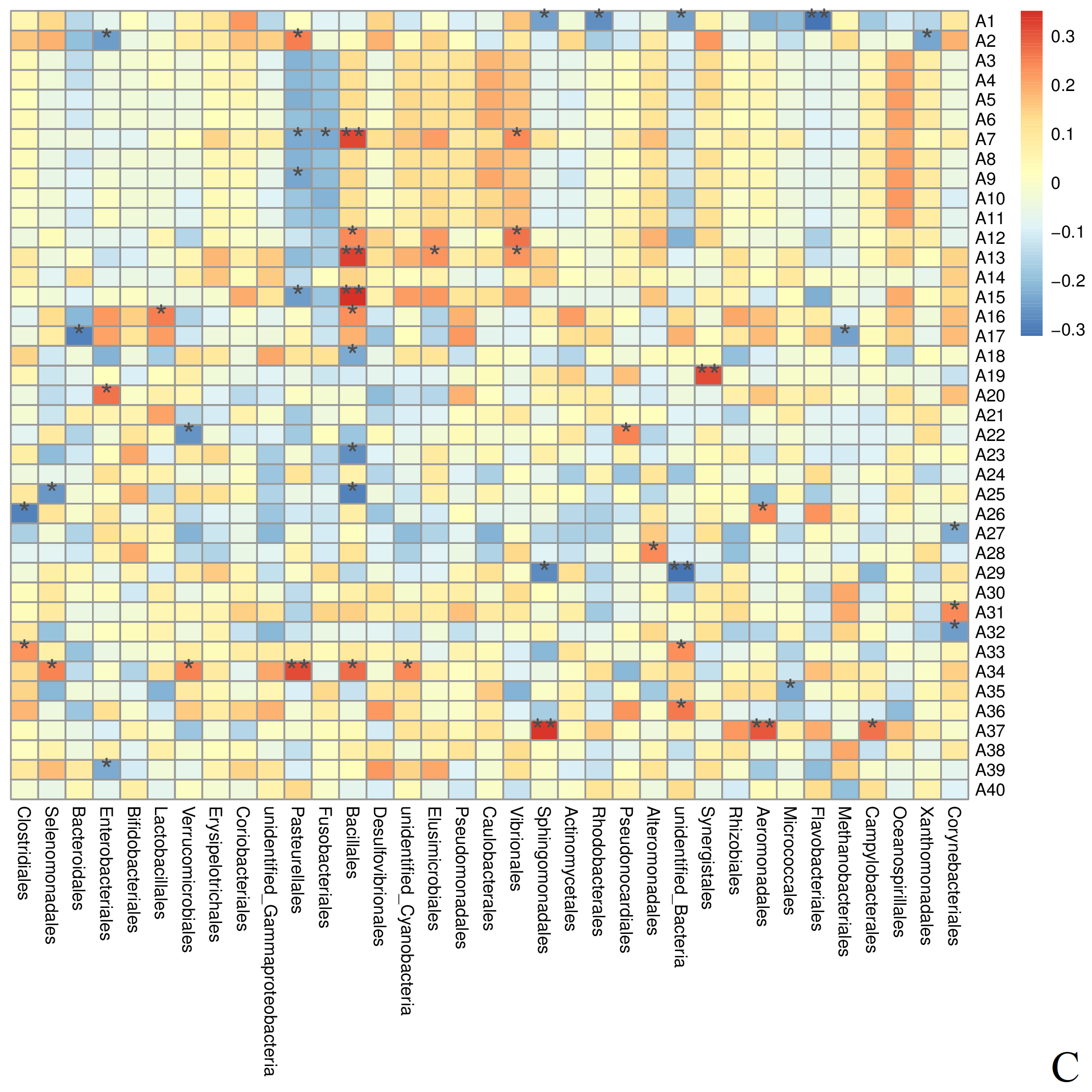

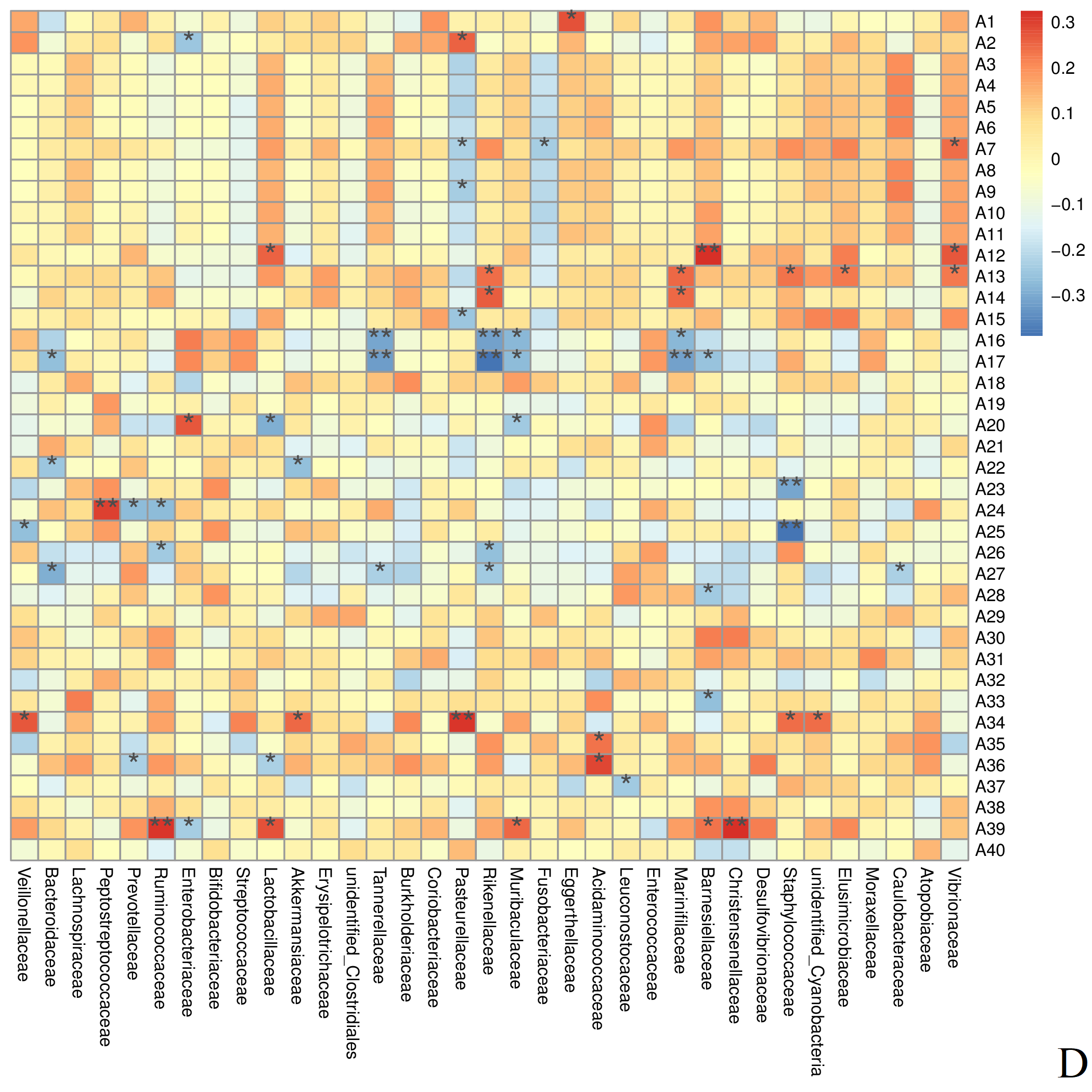

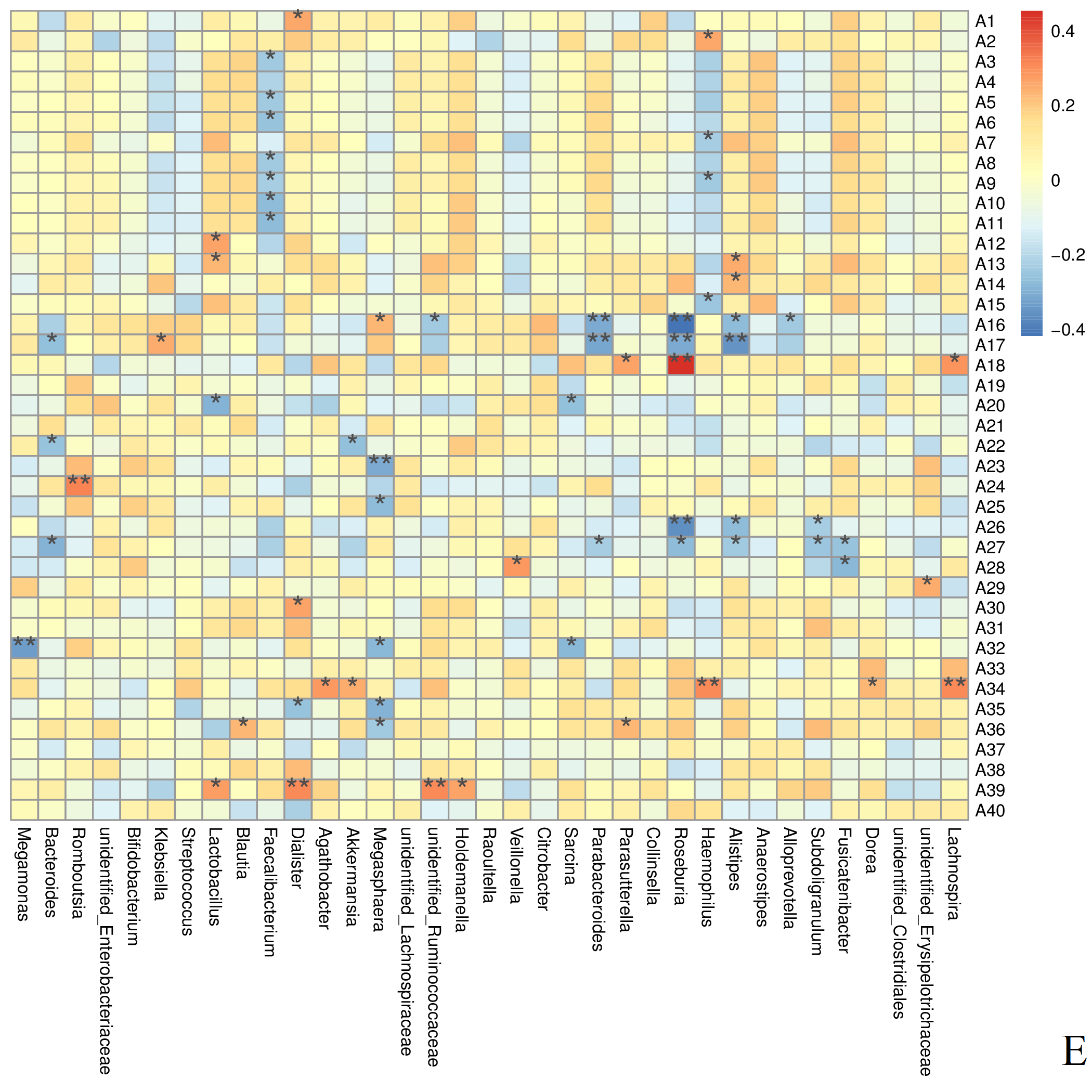

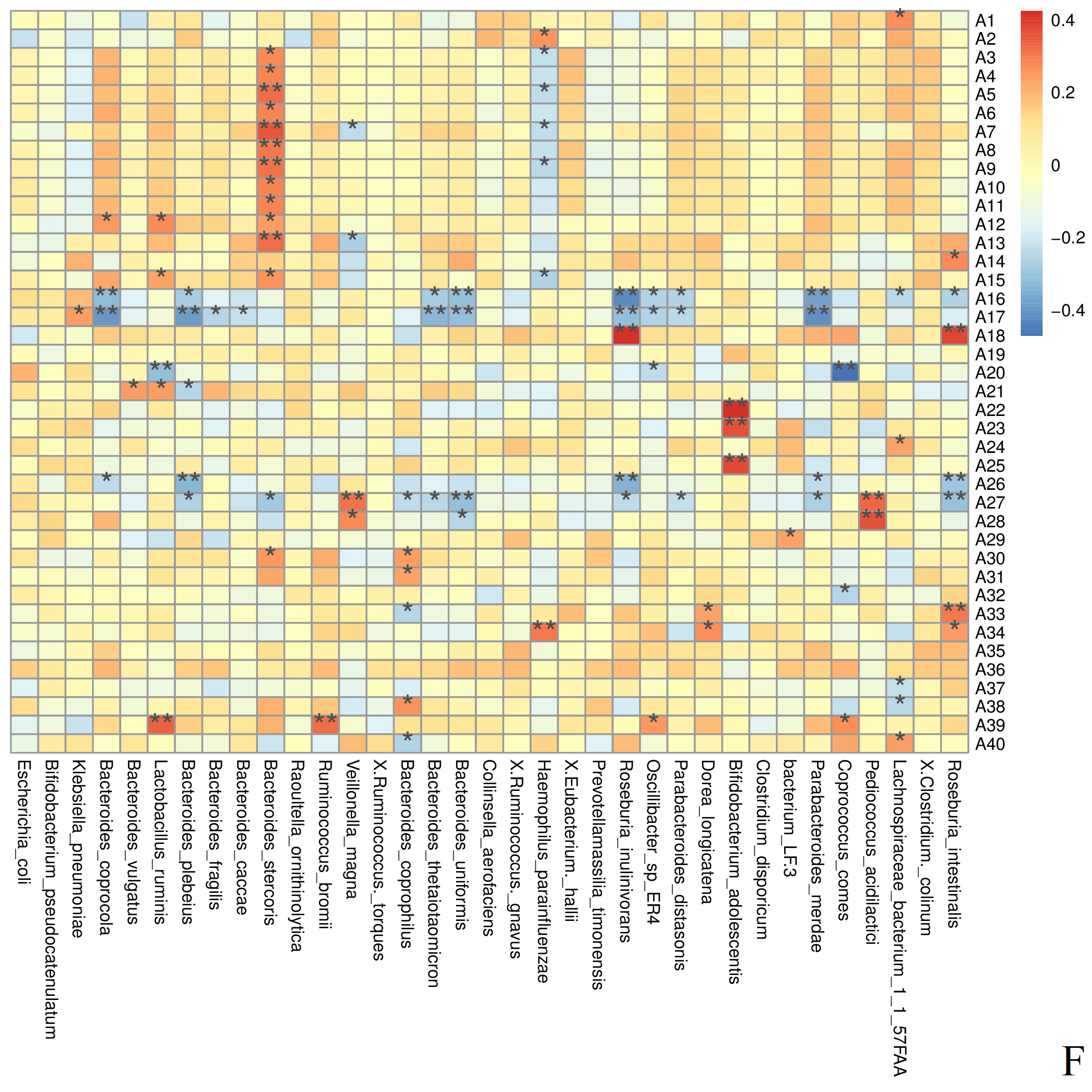


| Code | Detailed information |
| --- | --- |
| A1 | SBP, mmHg |
| A2 | DBP, mmHg |
| A3 | Intracellular Fluid, kg |
| A4 | Extracellular Fluid, kg |
| A5 | Protein, kg |
| A6 | Inorganic salt, kg |
| A7 | Body Fat Mass, kg |
| A8 | Basic metabolism, kcal |
| A9 | Total body water |
| A10 | Muscle Mass, kg |
| A11 | Lean Body Mass, kg |
| A12 | Weight, kg |
| A13 | Visceral fat, cm^2^ |
| A14 | Percent Body Fat, % |
| A15 | Waist-hip ratio, % |
| A16 | ALT, IU/L |
| A17 | AST, IU/L |
| A18 | AST/ALT |
| A19 | TBAC, μmol/L |
| A20 | Glu, mmol/L |
| A21 | UA, μmol/L |
| A22 | TG, mmol/L |
| A23 | Chol, mmol/L |
| A24 | HDL-C, mmol/L |
| A25 | LDL-C, mmol/L |
| A26 | HB, g/dl |
| A27 | HbA1c, g/dl |
| A28 | HbA1c/HB |
| A29 | CORT, μg/dl |
| A30 | Insulin, μU/mL |
| A31 | Serum C-peptide, ng/mL |
| A32 | Cu, μg/mL |
| A33 | Zn, μg/mL |
| A34 | Ca, μg/mL |
| A35 | Mg, μg/mL |
| A36 | Fe, μg/mL |
| A37 | Pb, μg/L |
| A38 | HOMA-IR |
| A39 | HOMA-β |
| A40 | HOMA-IS |

SBP, Systolic blood pressure; DBP, Diastolic blood pressure; ALT, Alanine aminotransferase; AST, Aspartate aminotransferase; TBAC, Total bile acid; HDL-C, High density lipoprotein cholesterol; LDL-C, Low density lipoprotein cholesterol; Glu, Glucose; UA , Uric acid; TG, Triglyceride; Chol, Cholesterol; CORT, Serum cortisol; HB, Hemoglobin; HbA1c, Glycosylated hemoglobin; HbA1c/HB, Glycosylated hemoglobin ratio; BMI, body mass index; HOMA, Homeostasis model assessment.

* p value<0.05, * *p value<0.01
